# CXCR4-targeted PET imaging of glioblastoma using [^68^Ga]Ga-TD-01: from pharmacokinetics and dosimetry to theranostic potential

**DOI:** 10.1186/s41181-026-00457-9

**Published:** 2026-05-21

**Authors:** Piyapan Suwattananuruk, Stine Figenschau, Angel Moldes-Anaya, Efat Muhammad Arshad, Rodrigo Berzaghi, Lars Kjelsberg Pedersen, Rune Sundset, Pornchai Rojsitthisak, Opa Vajragupta, Mathias Kranz

**Affiliations:** 1 PET Imaging Center, University Hospital of North Norway, Hansine Hansens Vei 82, 9019 Tromsø, Norway; 2 Department of Medical Biology, Tumor Biology Research Group, UiT The Arctic University of Norway, Hansine Hansens Vei 82, 9037 Tromsø, Norway; 3 Department of Clinical Medicine, UiT The Arctic University of Norway, Hansine Hansens Vei 82, 9037 Tromsø, Norway; 4 Department of Neurosurgery, Ophthalmology and Otorhinolaryngology, University Hospital of North Norway, Hansine Hansens Vei 82, 9019 Tromsø, Norway; 5 Department of Food and Pharmaceutical Chemistry and Center of Excellence in Natural Products for Ageing and Chronic Diseases, Faculty of Pharmaceutical Sciences, Chulalongkorn University, Phayathai Road, Pathumwan, Bangkok, 10330 Thailand; 6 Molecular Probes for Imaging Research Network, Faculty of Pharmaceutical Sciences, Chulalongkorn University, Phayathai Road, Pathumwan, Bangkok, 10330 Thailand

**Keywords:** CXCR4, Chemokine receptor PET imaging, Tumor microenvironment, PET pharmacokinetic modeling, Predictive dosimetry, Whole-body dosimetry, Pharmacokinetic, Glioblastoma

## Abstract

**Background:**

Glioblastoma multiforme (GBM) is an aggressive brain tumor with poor prognosis and limited treatment options. Chemokine receptor type 4 (CXCR4) plays a key role in GBM invasion and therapy resistance, making it an attractive target for molecular imaging and theranostics. We developed [^68^Ga]Ga-TD-01, a next-generation positron emission tomography (PET) radiotracer for CXCR4 imaging, and evaluated its pharmacokinetics, dosimetry, and translational potential.

**Results:**

In mice bearing orthotopic GL261 GBM, dynamic PET/MRI, receptor blocking studies, and kinetic modeling demonstrated a specific binding component and high tumor-to-background contrast. [^68^Ga]Ga-TD-01 showed high tumor uptake (5.4 ± 4.3%ID/g at 9 min), low uptake in normal brain (2.2 ± 1.7%ID/g), and a tumor-to-background ratio of 2.9 ± 0.2 at 60 min, which was significantly reduced by CXCR4 blockade. PET pharmacokinetic modeling confirmed increased tumor retention (Vt of 0.5 ± 0.07 ml/cm^3^ for tumor; Vt of 0.2 ± 0.05 ml/cm^3^ for brain). The radiotracer exhibited high in vivo stability, rapid renal clearance, and favorable biodistribution. CXCR4 expression was confirmed in both murine and human GBM tissues by RNAscope. Human dosimetry extrapolation estimated an effective dose of 4.6 mSv for a standard PET scan, comparable to approved tracers.

**Conclusions:**

When compared with reported data for [^68^Ga]Ga-Pentixafor, [^68^Ga]Ga-TD-01 showed tumor uptake within the reported range and low background accumulation; however, no direct head-to-head comparison was performed. These results support [^68^Ga]Ga-TD-01 as a promising CXCR4-targeted imaging agent and a candidate for further theranostic development.

**Supplementary Information:**

The online version contains supplementary material available at 10.1186/s41181-026-00457-9.

## Background

Glioblastoma multiforme (GBM) is an aggressive, treatment-resistant primary brain tumor characterized by poor clinical outcomes and limited therapeutic options (Wu et al. 2021). One crucial factor in GBM progression is the overexpression of chemokine receptor 4 (CXCR4) (Stevenson et al. 2008) involved in tumor growth, angiogenesis, and metastasis (Domanska et al. 2013). CXCR4 is highly expressed in GBM cells (Zhou et al. 2014), making it a valuable target for both diagnostic imaging and therapeutic interventions. However, conventional imaging modalities such as magnetic resonance imaging (MRI) and computed tomography (CT) provide insufficient insight into the molecular characteristics of GBM (Omuro et al. 2006). Positron emission tomography (PET) imaging, particularly using CXCR4-specific radiotracers, could improve patient selection and tumor characterization, allowing for more personalized treatment approaches.

CXCR4-specific PET radiotracers such as [^68^Ga]Ga-Pentixafor and [^177^Lu]Lu-Pentixather are currently used for imaging or treatment in various cancers, including gliomas (Lindenberg et al. 2024; Jacobs et al. 2022a; Buck et al. 2022). While [^68^Ga]Ga-Pentixafor has shown potential in targeting CXCR4, it presents significant pitfalls. One limitation is its variable tumor uptake, which does not consistently correlate with CXCR4 expression levels in GBM (Jacobs et al. 2022a), thereby reducing its utility for accurate patient selection (Buck et al. 2022). This inconsistent uptake can lead to false-negative results, limiting its ability for assessing CXCR4 status in clinical settings. Moreover, off-target binding and uptake in non-tumor tissues results in high background signals, further complicating image interpretation and reducing the overall diagnostic utility of the radiotracer (Serfling et al. 2022).

To address these limitations, the development of new PET radiopharmaceuticals is essential. A promising candidate was recently developed based on TIQ15 (Truax et al. 2013a), [^68^Ga]Ga-TD-01, a radiopharmaceutical designed for high-affinity binding to CXCR4 (Suwattananuruk et al. 2024). It was demonstrated that [^68^Ga]Ga-TD-01 targets CXCR4-positive GBM with evidence of a specific binding component, making it a potential candidate for targeted imaging of GBM tumors. The ability to differentiate between CXCR4-positive and CXCR4-negative tumors may facilitate the identification of patients who could benefit from CXCR4-targeted therapeutic interventions.

In theranostics, the [^68^Ga]/[^177^Lu] combination represents a well-established model, however, the use of different metals and therefore different metal coordination chemistry, introducing significant differences in pharmacokinetics between diagnostic and therapy applications (Wang et al. 2025). In contrast, the [^64^Cu]/[^67^Cu] true theranostic pair preserves the chelation chemistry and molecular identity when applied to imaging or therapeutic protocols, thereby preserving the in vivo behavior of the radiolabeled molecule (Søndergaard et al. 2024). Although the [^64^Cu]/[^67^Cu] pair is conceptually ideal it has also some limitations: i) the production process of Cu-64 is technically challenging, with few sites producing it worldwide, ii) the cost of the production process is high, as it requires isotopically enriched Ni-64 targets and advanced specialized equipment for production. Likewise, production of Cu-67 is technically challenging and expensive due to the need for enriched Zn-70 in its production process. However, it is possible to produce Cu-67 in biomedical cyclotrons (Søndergaard et al. 2024) with production yields suitable for exploratory preclinical studies (Ekaney et al. 2025). Future wider access to radiocopper isotopes will enhance the possibility of a more precise theranostic approach as compared to current strategies using [^68^Ga]/[^177^Lu].

In this study, we investigate the pharmacokinetic profile, in vivo stability and whole-body radiation dosimetry of the CXCR4-specific radiopharmaceutical [^68^Ga]Ga-TD-01 and provide tumor absorbed dose estimates of a potential copper-67 labeled TD-01 therapeutic application in GBM. This theranostic approach may enhance GBM management by enabling precise tumor detection, patient selection, and monitoring of treatment response, while potentially supporting the development of targeted radiotherapy for CXCR4-expressing tumors.

## Materials and methods

### Animals and ethics

Animal experiments were approved by the respective Norwegian authorities (Mattilsynet, FOTS ID 28409/30665) and conducted under national and international ethical standards. 29 six- to eight-week-old female C57BL/6JRj mice 21.7 ± 0.8 g (n = 6 whole-body dosimetry, n = 6 tumor development, n = 7 receptor blocking studies, n = 10 ex vivo stability) were purchased from Janvier (Le Genest-Saint-Isle, France) and housed 5 days for acclimatization before start of experiments. The orthotopic tumor cell inoculation of GL261 cells to create the animal model of brain cancer was performed according to our previous studies (Suwattananuruk et al. 2024; Lindemann et al. 2023). Following three to four weeks of tumor growth PET experiments were performed when the tumor reached a size of 0.09 ± 0.05 (0.04–0.18) cm^3^, measured by weekly MRT monitoring. RNAscope analysis was performed on tissue from 13 tumor-bearing animals included in the current study together with 5 additional subjects from previous experiments.

The use of human GBM tissue was approved by the regional ethical committee (REK) under the ID 295739.

### RNAscope tissue staining

For CXCR4 expression analysis, brains from 18 C57BL/6JRj mice bearing GL261 tumor were fixed in 4% formaldehyde solution (1.00496, Sigma-Aldrich) and paraffin-embedded (FFPE). Manual chromogenic in situ hybridization was performed on FFPE sections using the RNAscope® 2.5 HD Reagent Kit (322,310, Advanced Cell Diagnostic (ACD)) in accordance with the manufacturer’s instructions. Briefly, specimens were cut into 4 µm thick sections, incubated at 60 °C for 45 min, deparaffinized, and dehydrated. For tissue pretreatment, sections were exposed to hydrogen peroxide (322,330, ACD) for 15 min at room temperature, incubated in Target Retrieval solution (322,000, ACD) for 15 min at 97–98 °C and treated with Protease Plus solution (322,330, ACD) for 30 min at 40 °C in the HybEZ Oven® (PN321710, ACD). Hybridization of the CXCR4 probes (Mm-Cxcr4 #425,901, Hs-CXCR4 #310,511, ACD) was performed by incubation in the HybEZ® Oven for 2 h at 40 °C followed by standard signal amplification steps. Sections were incubated with DAB solution (322,310, ACD) for 10 min at room temperature. Finally, sections were counterstained with hematoxylin (RALA361075, VWR), rehydrated in a series of ethanol, and coverslipped using Histokitt (21,412, Chemi-Teknik AS). Probes targeting PPIB and dapB were used as positive and negative controls, respectively. Consecutive sections were stained using a H&E staining kit (ab245880, Abcam). Brightfield images were acquired using a VS120 Virtual slide scanner microscope (Olympus Life Science) at 20 × magnification. For each slide, 1 × 1 mm ROIs were selected from representative tumor and non-tumor regions. DAB-positive staining was segmented using a pixel classifier trained in Fiji/ImageJ with Labkit (Arzt et al. 2022). The trained classifier was applied uniformly across all ROIs. DAB-positive area was quantified and normalized to total ROI area.

### Synthesis of [^68^Ga]Ga-TD-01

The radiopharmaceutical production of [^68^Ga]Ga-TD-01 was recently described by our group (Suwattananuruk et al. 2024). Additional data and information can be found in Table 1 and in the supplemental material (Supplementary Fig. 1–4 and Supplementary Table 1). Briefly, the radiochemistry production of [^68^Ga]Ga-TD-01 starts with the conjugation of the CXCR4 antagonist TIQ15 to the functionalized chelator p-NCS-Bz-DOTA, producing TD-01, a bifunctional chelator. This reaction was performed in ammonium acetate buffer (pH 8) and was followed by purification through solid phase extraction (SPE). Radiolabeling was subsequently performed by eluting gallium-68 (^68^Ga) from a ^68^Ge/^68^Ga generator (GalliaPharm generator, Eckert & Ziegler Radiopharma GmbH, Berlin, Germany) using 0.1 N HCl. The ^68^GaCl_3_ (~ 700 MBq) was added to a reaction vial containing TD-01 (50 µg) in ammonium acetate buffer (pH 4) and heated at 60 °C for 10 min to complete the radiolabeling process, forming [^68^Ga]Ga-TD-01. After cooling, the product was purified via an SPE cartridge, washed with water, and eluted with ethanol. The ethanol was removed, and the final product was formulated in saline for use. The radiochemical purity, which exceeds 99%, was confirmed by radio-TLC and HPLC, and the molar activity was determined to ensure its suitability for in vivo applications.

**Table 1:** Quality control of [^68^Ga]Ga-TD-01 (n = 4)

Test parameters	[^68^Ga]Ga-TD-01
Total synthesis time	45–50 min
Radiochemical purity (RCP)	> 97%
Radiochemical yield (RCY)	41.52 ± 3.20% (EOS)
Molar activity (A_m_)	51.14 ± 4.74 GBq/µmol (EOS)
Appearance	Colorless solution
Radionuclidic purity	> 99%
Formulation	Normal saline
pH	∼ 7

### PET/MR imaging

All imaging sessions of the female C57BL/6JRj mice were performed under isoflurane anesthesia (induction 4%, maintenance 1.8% in O_2_). The mice were placed on a heated pallet (MINERVE, France) and respiratory signal recorded during the whole experiment. One-hour dynamic PET/MRI (7 T, PET/MRI, MR solutions, UK), n = 13 animals, 19.9 ± 1.4 g, was started simultaneously with an i.v. injection of 8.2 ± 1.5 MBq or 6.6 ± 1.5 MBq [^68^Ga]Ga-TD-01, dosimetry or tumor study, respectively. This time window was chosen to capture tracer distribution and clearance kinetics as well as stabilization of tumor uptake and background activity in normal brain, resulting in optimal tumor-to-background contrast. The list-mode data were reconstructed into 24 × 5 s, 8 × 60 s, 10 × 300 s time frames using 3D ordered subset expectation maximization with 1 iteration, 32 subsets, and a voxel size of 0.42 mm, applying correction for random coincidences, decay, deadtime, and scatter correction. The receptor blocking studies (n = 7) were performed by i.v. pre-injection of TIQ15 (10 mg/kg) ten minutes prior radiotracer injection.

A T2-weighted high resolution FSE sequence (TE = 45 ms, TR = 3000 ms, flip angle 90 degrees, 4 averages, voxel size x, y, z, 0.08, 0.6, 0.08 mm) using a dedicated brain coil was performed for subsequent tumor or brain segmentation. The hyperintense tumor region was manually segmented and a reference region was placed on the contralateral healthy hemisphere of the respective animal (PMOD, v.4.3, PMOD technologies, Switzerland), supplementary Fig. 5. Finally, the MRI data were superimposed with the dynamic PET and TAC expressed as %ID (Supplementary Fig. 1).

Whole body MRI was performed using a dedicated mouse body coil with T1-weighted FSE sequences (TE = 11 ms, TR = 1312 ms, flip angle 90 degrees, 4 averages, voxel size x, y, z, 0.24, 1.1, 0.24 mm).

### Dosimetry

Whole-body incorporation dosimetry was performed using time-activity data of six healthy female C57BL/6JRj mice. Brain, lungs, heart, liver, gallbladder, stomach, spleen, kidneys, small intestines, large intestines (LI) divided into upper large intestines (ULI) and lower large intestines (LLI), urinary bladder wall and remainder of body were segmented on T1-weighted MRI and superimposed with the dynamic PET data to extract the time-activity curve and % injected dose values (%ID) (PMOD, v.4.3, PMOD technologies, Switzerland). To account for metabolic differences between mice and human due to significant difference in body size and weight an allometric scaling was performed according to Eqs. 1 and 2 for %ID or time scale, respectively (Kirschner et al. 1975; Sparks and Aydogan 1999).

1 $$ \frac{{{{\% ID}}}}{{{\mathrm{organ}}_{{{\mathrm{human}}}} }}{ = }\frac{{{{\% ID}}}}{{{\mathrm{g}}_{{{\mathrm{animal}}}} }} \cdot {\mathrm{m}}_{{{\mathrm{organ}}_{{{\mathrm{human}}}} }} \cdot \frac{{{\mathrm{m}}_{{{\mathrm{animal}}}} }}{{{\mathrm{m}}_{{{\mathrm{human}}}} }} $$

with the fraction of the injected activity in the corresponding human organ = $\frac{\mathrm{\%ID}}{{\mathrm{organ}}_{\mathrm{human}}}$, the fraction of injected activity per gram animal organ tissue = $\frac{\mathrm{\%ID}}{{\mathrm{g}}_{\mathrm{animal}}}$ and ${\mathrm{m}}_{{\mathrm{organ}}_{\mathrm{human}}}$ the mass of the corresponding human organ (Valentin 2002).

2 $${\mathrm{t}}_{\mathrm{human}}\mathrm{=}{\mathrm{t}}_{\mathrm{animal}}{\left\{\frac{{\mathrm{m}}_{\mathrm{human}}}{{\mathrm{m}}_{\mathrm{animal}}}\right\}}^{0.25}$$

with the human time scale = ${\mathrm{t}}_{\mathrm{human}}$, the animal time scale =${\mathrm{t}}_{\mathrm{animal}}$ and the ratio of animal and human body weights = $\frac{{\mathrm{m}}_{\mathrm{animal}}}{{\mathrm{m}}_{\mathrm{human}}}$.

The scaled %ID based TACs were entered into the exponential modeling tool of OLINDA (v1.1 Vanderbilt University, Nashville, TN, USA) and bi- or tri-exponential functions fitted (least mean squares) to calculate the number of disintegrations (NOD). Organ doses (OD) and the effective dose (ED) were estimated for the adult male model. The ED contributions were calculated by multiplying the ODs with the tissue weighting factors of ICRP103 (Protection 2007).

### Predictive tumor dose for treatment with [^67^Cu]Cu-TD-01

The NOD were derived from dynamic PET with [^68^Ga]Ga-TD-01 using the kinetic modeling toolbox Pkin (PMOD, v.4.3, PMOD technologies, Switzerland) and the OLINDA exponential modeling toolbox (Vanderbilt University, Nashville, TN, USA, version 1.1). The mono-, bi- or tri- exponential functions were fitted optimizing the least squares. The TACs were corrected for differences in physical half-life between ^68^Ga and ^67^Cu as shown by others (Peters et al. 2022; Miller et al. 2022; Sgouros et al. 2004) before calculating the NOD. The dose calculation was performed using the sphere model of IDAC-dose 2.1 (Andersson et al. 2017) while the tumor volume was extracted from the MRI image (Supplementary Fig. 5). The tissue density was set to 1.05 g/cm^3^ representing closest the circumstances in brain tumor tissue (Lee et al. 2015).

### PET pharmacokinetic modeling

The TAC of brain tumor and brain (VOI in contralateral healthy hemisphere) were extracted and curves expressed as kBq/cm^3^. Due to the technical limitations of arterial blood sampling in mice, an image-derived input function (IDIF) was extracted from the inferior vena cava in early PET images (5 s post-injection) as shown in the supplementary Fig. 5. It provides a robust estimation of the IDIF and has been validated by others (Espedal et al. 2021; Lanz et al. 2014; Kuttner et al. 2024). Subsequently, the pharmacokinetic modeling was performed using the:

(i) 1-tissue-compartment model (1TCM) as a baseline model to describe tracer delivery and reversible tissue uptake, which is commonly used for radiotracers with fast kinetics and limited trapping.
(ii) 2-tissue-compartment model (2TCM) to additionally evaluated to account for a potential specific binding component, including receptor interaction and internalization, which is relevant for CXCR4-targeted tracers.
(iii) Logan graphical analysis as a model-independent approach to estimate distribution volume (Vt) without relying on compartmental assumptions, thereby providing complementary validation of the kinetic results.

The suitability of each model was evaluated by Schwarz Criterion (SC), Akaike Information Criterion (AIC) and Model Selection Criterion (MSC). The kinetic rate constants K1, k2, k3, k4 and the volume of distribution (Vt) were calculated. Finally, the baseline and receptor blocking data were compared to each other based on differences in rate constants and percent injected dose (%ID/g).

### Ex vivo stability of [^68^Ga]Ga-TD-01

The ex vivo stability of [^68^Ga]Ga-TD-01 was assessed in healthy female C57BL/6JRj mice (n = 10) at different time points, from 30 min up to two hours post-injection (n = 2–3). Each mouse received an intravenous injection of [^68^Ga]Ga-TD-01 via the lateral tail vein (63.5 ± 23.4 MBq). At the designated time points, blood samples (600–1000 μL) were collected and plasma separated by centrifugation. Subsequently, the radiolabeled compound was extracted from the plasma using 400 μL of 0.1% trifluoroacetic acid (TFA) in methanol. Plasma samples were centrifuged at 14,500 rpm at 4 °C for 4 min and the resulting supernatant was collected and analyzed for intact radioligand. Samples were analyzed by radio-HPLC with a gradient consisting of 0.1% TFA in ACN and 0.1% TFA in MilliQ water at a flow rate of 1 mL/min. The following elution timeline was applied: 0–2 min: 15% of 0.1% TFA in ACN, 2–5 min: 20% of 0.1% TFA in ACN, 5–7 min: 30% of 0.1% TFA in ACN, 7–10 min: 40% of 0.1% TFA in ACN, 10–17 min: 50% of 0.1% TFA in ACN, and 17–18 min: 15% of 0.1% TFA in ACN. The analysis was performed using a C18 HPLC column (XBridge C18, 4.6 × 150 mm, 5 μm, Waters Corporation, USA). For the latest time points (90 and 120 min), samples were fractionated after column elution and the collected fractions were analyzed by gamma counting.

### Statistics

Statistical data analysis was performed with GraphPad Prism (version 10.3.1 for Mac, GraphPad Software, Boston, Massachusetts, USA). Furthermore, a priori and post hoc power analysis was performed with G*Power (Faul et al. 2009) (Supplementary Material, Sect.  3). The numerical values are presented as mean ± standard deviation or median. The PET pharmacokinetic modeling data and RNAscope data were analyzed by an unpaired, two-sided t-test. A two-way ANOVA was performed to evaluate the effects of baseline and receptor blocking conditions on tumor and brain radiotracer uptake (measured as %ID/g) across multiple time points applied to the dynamic PET data. The analysis assessed the interaction between time (Row Factor) and treatment condition (Column Factor), as well as the main effects of each factor. Statistical significance was considered when the *p*-value was < 0.05 (95% confidence level).

## Results

### In vitro analysis of CXCR4 expression in animal and human tissue

**Fig. 1:**
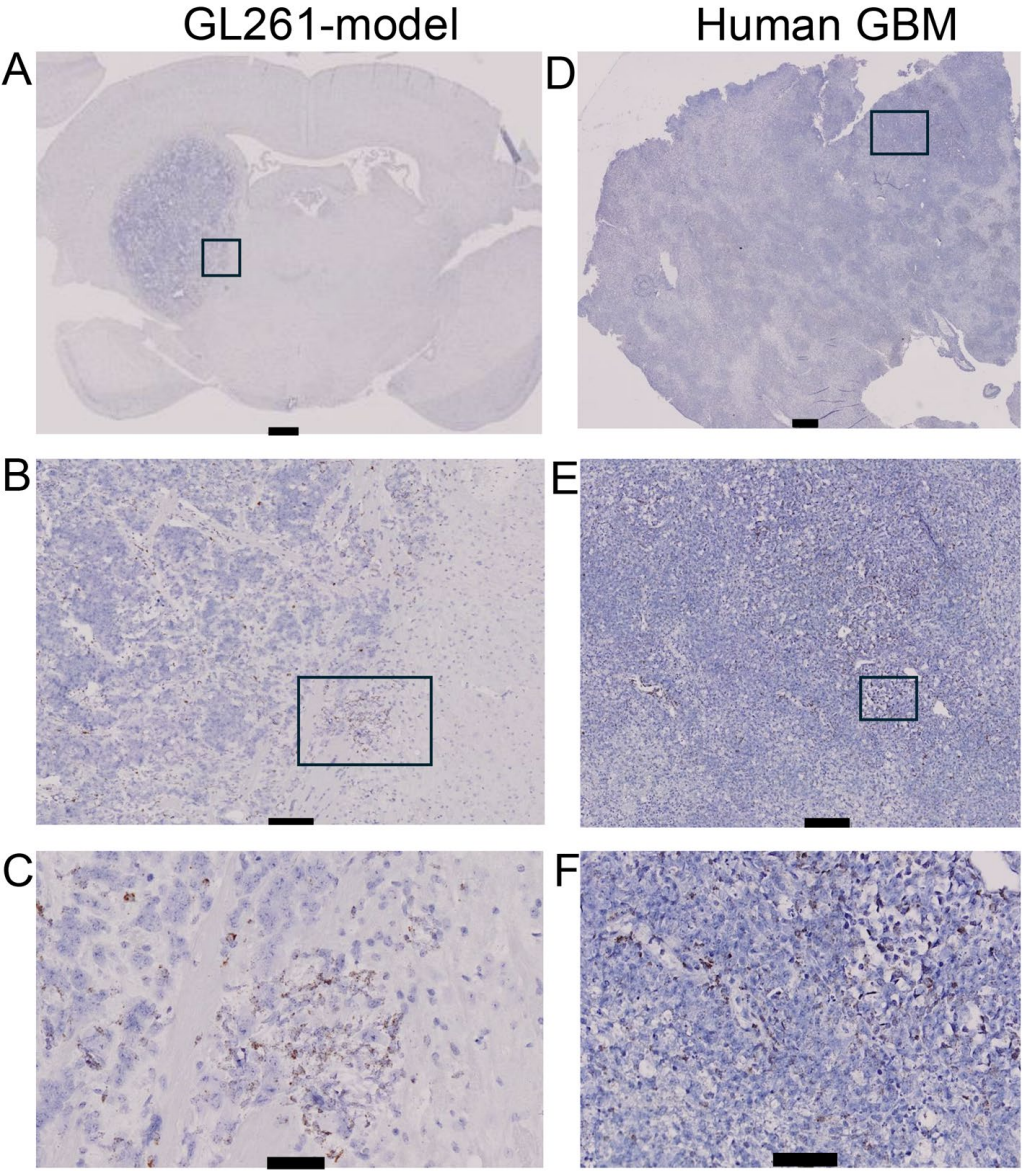
CXCR4 expression in murine and human GBM tissue. Left panel (mouse) (**A**–**C**) RNAscope in situ hybridization of GL261 mouse brain tumors showing intratumoral CXCR4 mRNA (brown puncta) expression, with no signal in adjacent healthy brain tissue. Right panel (human). (**D**–**F**) Corresponding CXCR4 mRNA expression in human GBM samples, confirming translational relevance. In panels A and D, the black box outlines the region shown at higher magnification in panels **B**, **C** and **E**, **F**, respectively, highlighting the cellular localization of CXCR4 transcripts. Scale bars: A, D = 1 mm; B, E = 600 µm; C, F = 60 µm

To assess CXCR4 mRNA expression in GBM tissue, RNAscope in situ hybridization was performed on both mouse (GL261 model, n = 18) and human GBM samples (Fig. 1A–F, Supplementary Fig. 8). Clear and specific punctate staining was observed within the tumor regions in both species, while adjacent non-tumor brain tissue remained unstained (mice), indicating low or absent CXCR4 expression in healthy brain parenchyma (Fig. 1A–C). Quantification of the DAB-positive area (Supplementary Fig. 8) revealed consistently elevated tumor-associated signal across all analyzed animals, resulting in substantially increased tumor-to-brain expression ratios. Importantly, these findings corresponded with the elevated tumor-to-brain contrast observed in PET imaging, supporting that the in vivo tracer accumulation reflects enhanced CXCR4 expression within the tumor microenvironment. Notably, inter-individual variability in CXCR4 expression was observed across tumors, consistent with the known biological heterogeneity of GBM and potentially contributing to the variability in tracer uptake observed in vivo. Similarly, patient-derived GBM samples showed distinct CXCR4-positive mRNA transcripts within tumor regions, confirming translational relevance.

### Dynamic PET/MRI and receptor blocking studies

**Fig. 2:**
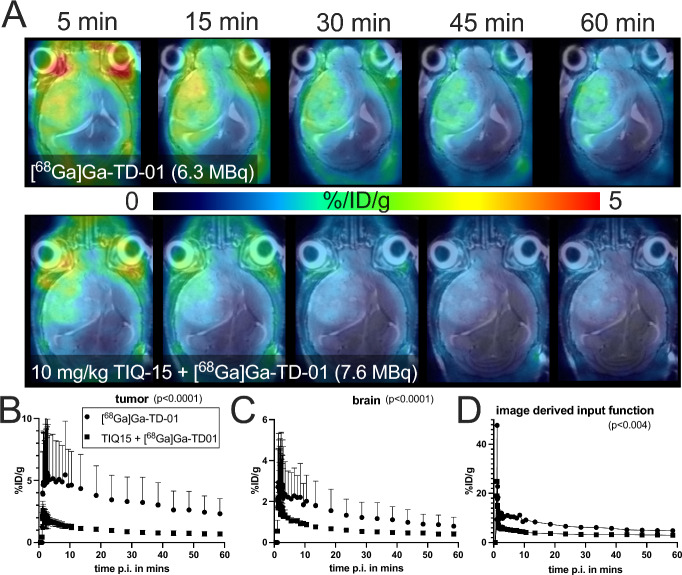
(**A**) Representative PET/MRI time series of baseline (upper panel) or receptor blocking study (lower panel) following i.v. injection of [^68^Ga]Ga-TD-01 only or by pre-blocking with TIQ15 (10 mg/kg). (**B**) Tumor TAC for baseline and blocking condition showing significant (t-test, *p* < 0.0001) lower uptake of [^68^Ga]Ga-TD-01in tumor following pre-blocking with TIQ15. C) Healthy brain TAC following baseline and blocking conditions with significant lower uptake of [^68^Ga]Ga-TD-01 after receptor pre-blocking with TIQ15 (t-test, *p* < 0.0001). D) Input function (mean) based on time activity data derived from vena cava. (**B**–**D**) Data are expressed as %ID/g and presented as mean ± SD (n = 13)

The PET/MRI time series (Fig. 2A) presents clear and distinct uptake of [^68^Ga]Ga-TD-01 in the tumor region for both baseline and blocking conditions. While radiotracer uptake is distinct over the whole 60-min time frame in the baseline studies, it is less pronounced in receptor blocking studies. The area under the curve following receptor blocking is more than threefold lower in the tumor region (199 vs. 58%ID/g·min) or twofold lower in healthy brain (79 vs. 35%ID/g·min). Accordingly, the TACs in Fig. 2B describes a marked reduction in uptake following receptor blocking of the tumor region while it’s less pronounced in the brain region as presented in Fig. 2C. The tumor-to-background ratio using the contralateral healthy brain region as reference presents its highest value 60 min post-injection with 2.9 ± 0.2.

Following statistical analysis using a two-way ANOVA test in the tumor region, the interaction between time and treatment in the tumor region was significant [F(42, 168) = 1.571, *p* = 0.0241], indicating that the effect of treatment (baseline versus blocking) was different across time points. Furthermore, there was a significant main effect of treatment [F(1, 4) = 8.9, *p* < 0.04], with blocking showing significantly lower tumor uptake (mean = 1.65%ID/g) compared to baseline (mean = 4.21%ID/g), a difference of 2.56. Additionally, the main effect of time was also significant [F (42, 168) = 7.085, *p* < 0.0001], suggesting variation in tumor uptake over the different time points. Resultantly, receptor blocking significantly reduced tumor uptake, with a time-dependent effect.

In the brain region, the interaction between time and treatment was not significant [F(1.499, 5.994) = 0.6277, *p* = 0.52], indicating that the effect of the treatment (baseline versus blocking) was consistent across time points. However, there was a significant main effect of time [F(1.499, 5.994) = 10.05, *p* < 0.0148], indicating that brain uptake varied significantly across time points. Additionally, the main effect of treatment (baseline versus blocking) was not significant [F(1, 4) = 1.06, *p* = 0.36]. Blocking resulted in significantly lower brain uptake (mean = 1.21%ID/g) compared to baseline (mean = 2.04%ID/g), with a mean difference of 0.83.

### Whole-body biodistribution and dosimetry

**Fig. 3:**
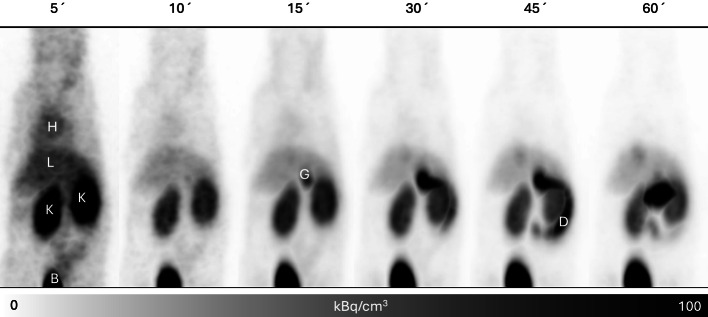
Representative time series (MIP) of one mouse after i.v. injection of 7.6 MBq [^68^Ga]Ga-TD-01 showing kidneys (K), liver (L), bladder (B), heart (H), gallbladder (G) and duodenum (D). Time-activity curves were subsequently extracted for dose calculation with OLINDA

**Fig. 4:**
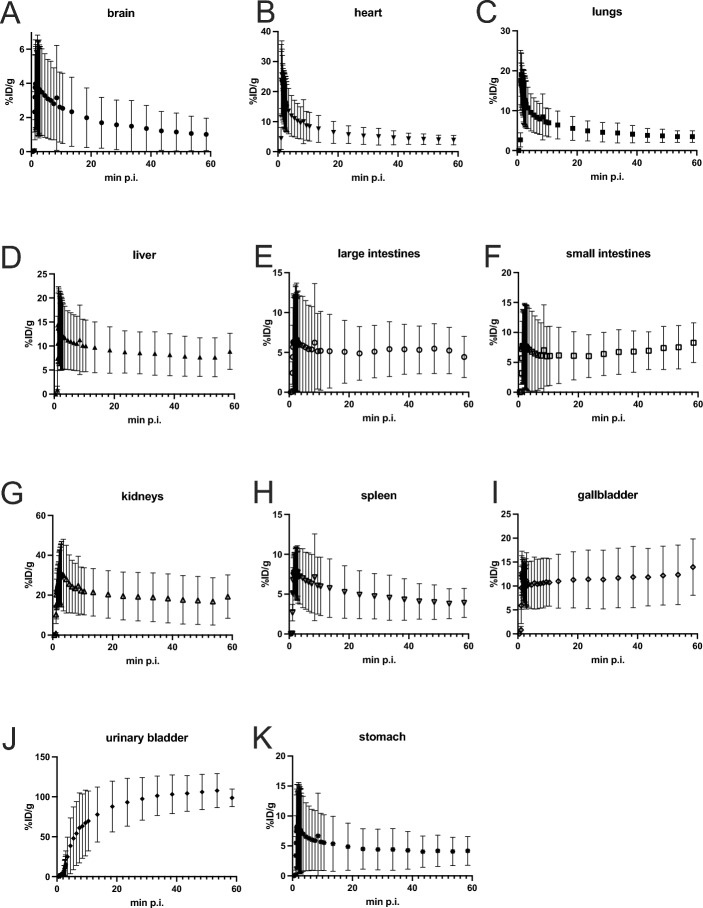
Time–activity curves derived from PET imaging showing the biodistribution of [^68^Ga]Ga-TD-01 in major organs. (**A**–**K**) Percentage of injected dose per gram (%ID/g) over time in brain, liver, kidneys, urinary bladder, heart, large intestine, spleen, stomach, lungs, small intestine, and gallbladder. The tracer demonstrates rapid blood clearance and predominant renal excretion, characterized by high kidney uptake and progressive accumulation in the urinary bladder. Low uptake is observed in normal brain tissue throughout the imaging period. Data are presented as mean ± SD

The in vivo biodistribution of [^68^Ga]Ga-TD-01 was assessed using dynamic PET imaging (Figs. 3, 4). The tracer exhibited rapid blood clearance and a predominantly renal excretion profile, as evidenced by high initial uptake in the kidneys followed by progressive accumulation in the urinary bladder. Kidney uptake peaked early and remained elevated over the imaging period, consistent with glomerular filtration and urinary elimination.

Uptake in the liver and gastrointestinal organs was moderate, indicating a minor hepatobiliary contribution to tracer clearance. Activity in the lungs and heart decreased rapidly over time, reflecting fast systemic distribution and clearance from the blood pool. Importantly, uptake in normal brain tissue remained consistently low throughout the study, supporting favorable imaging contrast for intracranial lesions.

Overall, the biodistribution profile of [^68^Ga]Ga-TD-01 is characterized by rapid systemic clearance, low retention in most tissues, and efficient renal elimination, which are desirable properties for PET imaging and dosimetry estimation.

**Fig. 5:**
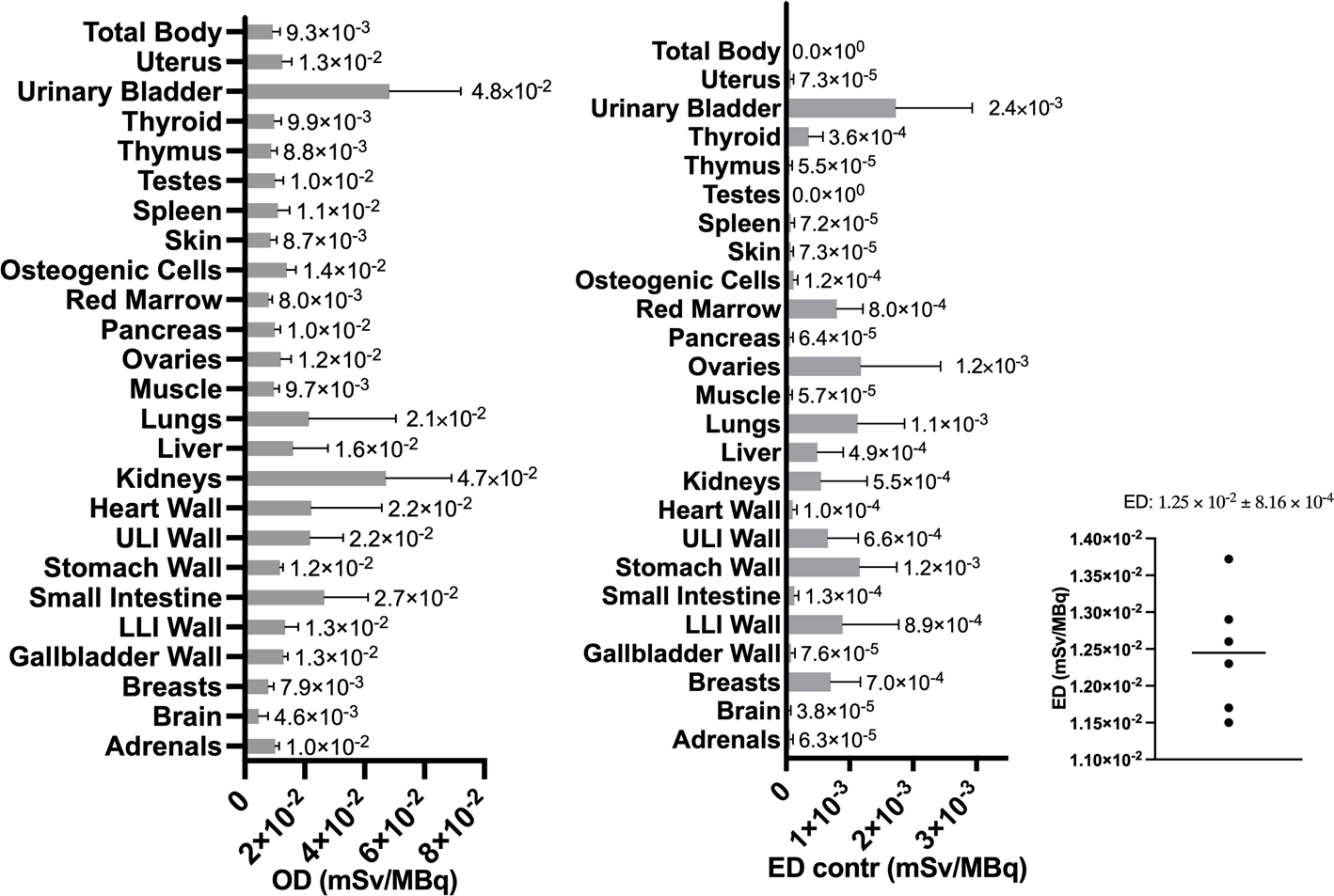
Organ doses (**A**), effective dose contributions (**B**) and effective dose (**C**) following application of 8.2 ± 1.5 MBq [^68^Ga]Ga-TD-01 and using biokinetic data from healthy mice (n = 6) extrapolated to humans and estimated by OLINDA v.1.1

To evaluate the safety and potential radiation burden of [^68^Ga]Ga-TD-01 following i.v. application the whole-body dosimetry in humans was estimated based on mouse biokinetic data (Fig. 4) and ODs and EDs calculations (Fig. 5, Supplementary Table 2). The highest OD (mean ± SD, mSv/MBq) was received by the urinary bladder wall 5.15 E-02 ± 2.5 E-02) and kidneys (5.01 E-02 ± 2.3 E-02) indicating a renal excretion pathway. Contrary, the brain received the lowest OD (5.29 E-03 ± 3.0 E-03).

Subsequently, including the tissue-specific weighting factors for the radiation risk, urinary bladder wall received the highest ED (mean ± SD, mSv/MBq) contribution (2.08 E-03 ± 9.50 E-04) followed by ovaries (1.42 E-03 ± 1.25 E-03) and stomach wall (1.40 E-03 ± 1.10 E-04). The overall radiation burden defined by the ED was 12.5 E-03 ± 8.16 E-04 μSv/MBq which equals 4.6 mSv for a typical PET scan using 370 MBq of radiotracer.

### Predictive tumor dosimetry

**Fig. 6:**
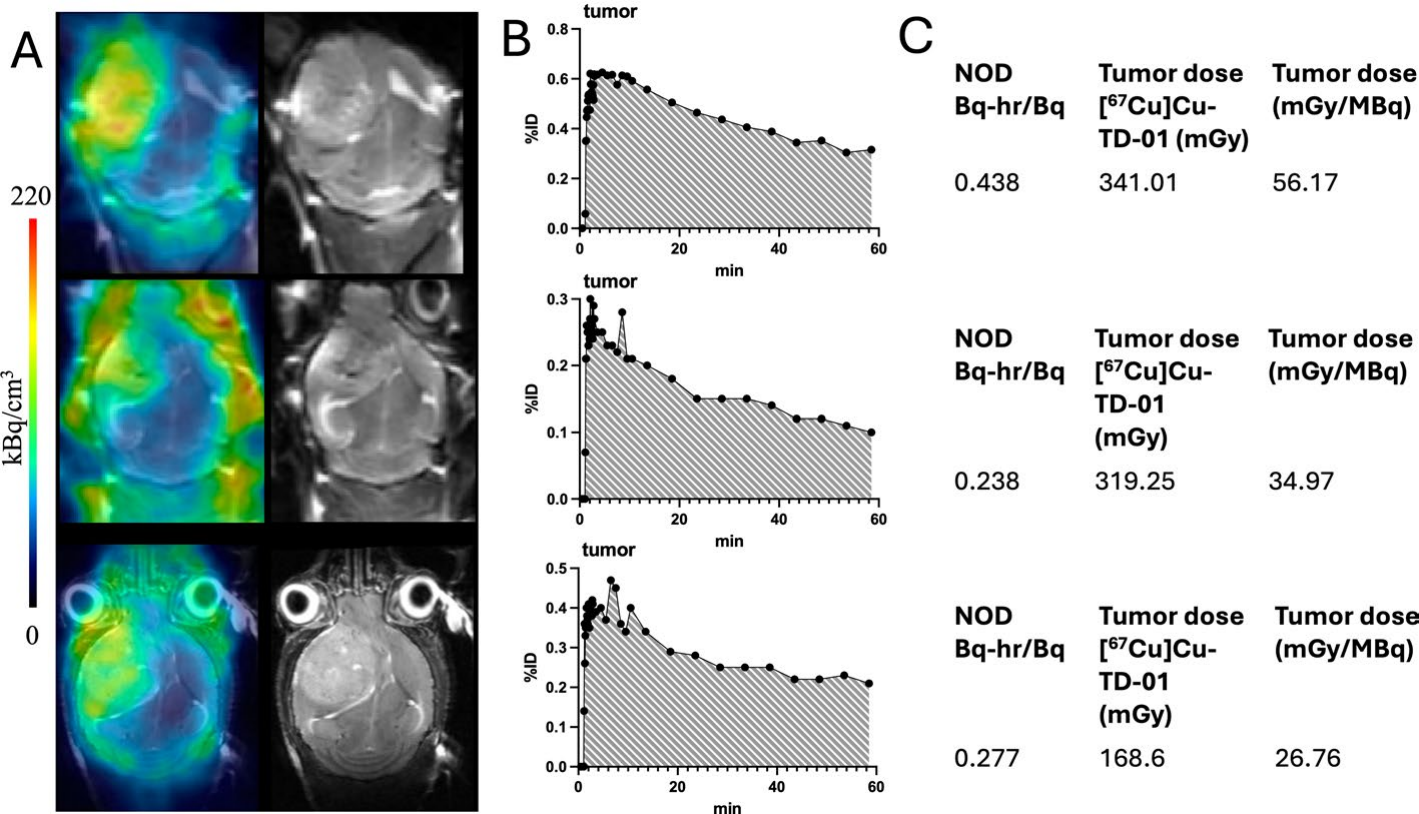
(**A**) PET/MRI of three representative tumor-bearing animals following i.v. injection of [^68^Ga]Ga-TD-01 and corresponding TAC (**B**). (**C**) Predictive tumor dose estimates based on [^68^Ga]Ga-TD-01 biokinetic data extrapolated to ^67^Cu in preparation for subsequent treatment studies. Of note, the tumor dose refers to the injection of [^68^Ga]Ga-TD-01 of 6.1, 9.1 or 6.3 MBq and tumor sizes of 0.12, 0.08 or 0.15 cm^3^ (animal top to down)

Predictive tumor dosimetry for a potential therapeutic application of TD-01 was performed using dynamic PET data acquired with [^68^Ga]Ga-TD-01 and extrapolated to the physical decay characteristics of ^67^Cu. Representative PET/MR images and corresponding TACs from three tumor-bearing animals are shown in Fig. 6A and B. Despite inter-animal variability in tumor size and injected activity, all tumors demonstrated sustained retention of [^68^Ga]Ga-TD-01 over the one-hour imaging period, supporting the feasibility of extrapolating these kinetics to a longer-lived therapeutic radionuclide. The derived NOD values translated into predicted absorbed tumor doses ranging from 168.6 to 341.0 mGy for the administered activities used in the PET studies (6.1–9.1 MBq), corresponding to an estimated tumor dose range of approximately 27–56 mGy/MBq. Smaller tumors with higher retention exhibited higher absorbed dose per MBq, highlighting the strong dependence of dosimetric outcome on lesion size and biological behavior.

### PET pharmacokinetic modeling of [^68^Ga]Ga-TD-01

**Fig. 7:**
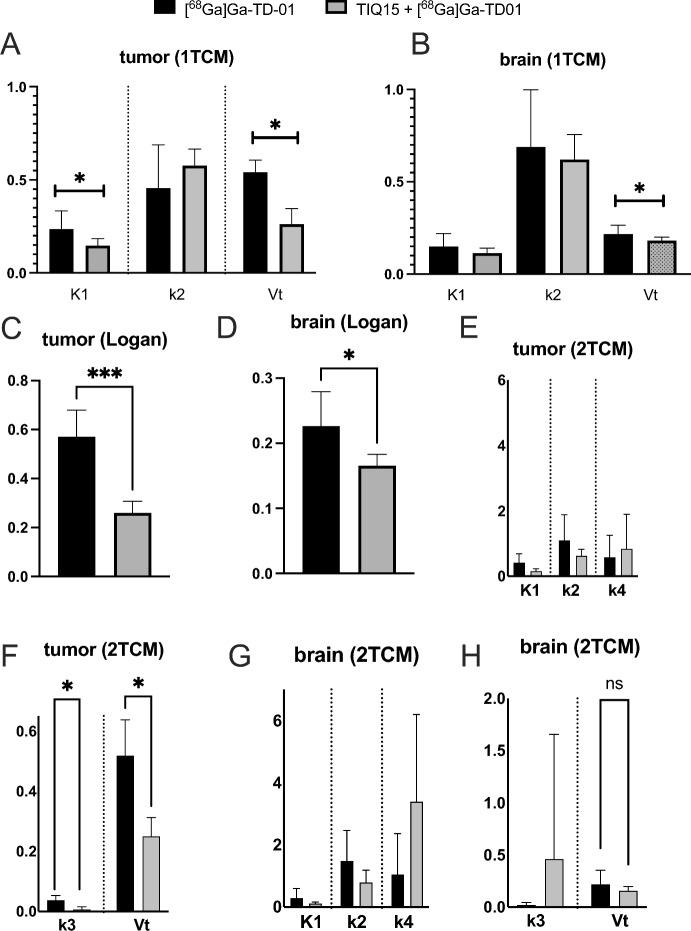
PET pharmacokinetic modeling results under baseline versus receptor blocking conditions using 10 mg/kg TIQ15 followed by one hour PET/MRI. According to the selection criteria the 1-tissue-compartement model (1TCM) provided the best fitting results for the pharmacokinetics of [^68^Ga]Ga-TD-01 and describe statistically significant the blocking effect in tumor (**A**) and healthy brain (**B**) region. The graphical Logan analysis confirmed findings of the 1TCM with regard to Vt while significant receptor blocking effects were present in tumor region (**C**) but not within the healthy brain tissue of the contralateral hemisphere (**D**). (**E**–**H**) Two-tissue compartment model analysis of [^68^Ga]Ga-TD-01 kinetics in tumor and brain under baseline and CXCR4-blocked conditions. (**E**) Estimated rate constants (K1, k2, k4) in tumor tissue, showing increased variability under blocking conditions. (**F**) k3 and total distribution volume in tumor, demonstrating a significant reduction upon CXCR4 blockade, consistent with a specific binding component. (**G**) Estimated rate constants (K1, k2, k4) in brain tissue. (**H**) k3 and Vt in brain, showing no significant changes between baseline and blocked conditions. Data are presented as mean ± SD; statistical significance indicated as *p < 0.05, **p < 0.01, ns = not significant.. K1: ml/cm^3^/min, k2: 1/min, k3: 1/min, k4: 1/min, Vt: ml/cm.^3^

Dynamic PET data were analyzed using both one-tissue (1TCM) (Fig. 7A, B and Table 2) and two-tissue (2TCM) (Fig. 7E–H and Table 3) compartment models to estimate kinetic parameters (K1, k2, k3, k4) describing tracer delivery, binding, and clearance. Parameter estimation was performed by nonlinear least-squares fitting using PMOD software (version 4.3). To determine the most appropriate model for [^68^Ga]Ga-TD-01 kinetics, model selection was guided by the Akaike Information Criterion (AIC) and the Bayesian Information Criterion (BIC, also referred to as the Schwarz Criterion, SC), which penalize model complexity. Both indices favored the 1TCM, as the 2TCM yielded less than a 5% improvement in residual variance while introducing additional free parameters, indicating that the simpler model adequately described the tracer kinetics.

**Table 2:** PET pharmacokinetic modeling results using the 1TCM and Logan (values in parentheses) analysis under baseline and blocking conditions

	K1 baseline		K1 blocking		k2 baseline		k2 blocking		Vt baseline		Vt blocking	
	Mean	SD	Mean	SD	Mean	SD	Mean	SD	Mean	SD	Mean	SD
Tumor	0.237	0.097	0.15	0.036	0.456	0.233	0.577	0.089	0.541 (0.57)	0.065 (0.11)	0.262 (0.26)	0.083 (0.05)
Brain	0.150	0.070	0.113	0.028	0.689	0.309	0.621	0.134	0.217 (0.23)	0.048 (0.05)	0.182 (0.17)	0.017 (0.02)

**Table 3:** PET pharmacokinetic modeling results using the 2TCM under baseline and blocking conditions. Mean ± stdev

	K1	K1 block	k2	k2 block	k3	k3 block	k4	K4 block	Vt	Vt block
Tumor	0.41 ± 0.27	0.16 ± 0.07	1.1 ± 0.79	0.62 ± 0.21	0.04 ± 0.02	0.007 ± 0.009	0.58 ± 0.68	0.832 ± 1.07	0.52 ± 0.12	0.25 ± 0.06
Brain	0.29 ± 0.31	0.11 ± 0.05	1.50 ± 0.97	0.79 ± 0.40	0.02 ± 0.02	0.46 ± 1.2	1.05 ± 1.32	3.39 ± 2.81	0.22 ± 0.14	0.16 ± 0.04

The goodness-of-fit was evaluated by the AIC, SC and MSC while the 1TCM and Logan analysis delivered the best fitting results (Supplementary Fig. 6). Following PET pharmacokinetic modeling, the kinetic parameters (mean ± SD) for the tumor using the 1TCM model, including K1 (ml/cm^3^/min), k2 (1/min), and Vt (ml/cm^3^), were significantly altered by the co-administration of TIQ15 (Fig. 7A, Table 1). The 1TCM model and Logan plot delivered the best estimates for the kinetic parameters of [^68^Ga]Ga-TD-01. For the [^68^Ga]Ga-TD-01 group, K1 was observed to be 0.24 ± 0.097 ml/cm^3^/min, k2 was 0.46 ± 0.23 1/min, and Vt was 0.54 ± 0.07 ml/cm^3^. Upon co-administration of TIQ15, K1 decreased to 0.15 ± 0.04 ml/cm^3^/min (*p* < 0.05), while k2 increased to 0.57 ± 0.1 1/min, and Vt significantly decreased to 0.26 ± 0.08 ml/cm^3^ (*p* < 0.05) indicating a competitive inhibition at the receptor site.

In addition to compartmental modeling, Logan graphical analysis was performed to obtain model-independent estimates of the total distribution volume. The Logan graphical analysis (Fig. 7C) yielded similar results, reinforcing the observations from the 1TCM model (Fig. 7A) and similarly demonstrated a reduction upon CXCR4 blockade in tumor tissue. In the [^68^Ga]Ga-TD-01 group, the tumor Vt was measured at 0.57 ± 0.11 ml/cm^3^, which significantly decreased to 0.26 ± 0.05 ml/cm^3^ (*p* < 0.05) in the presence of TIQ15.

In contrast to the tumor results, the kinetic parameters for the healthy brain region displayed no significant difference for K1 which decreased from 0.15 ± 0.07 ml/cm^3^/min to 0.13 ± 0.03 ml/cm^3^/min while k2 remained stable at 0.69 ± 0.31 1/min or 0.71 ± 0.19 1/min under baseline versus blocking conditions, respectively. In contrast, Vt presented significant (*p* < 0.05) differences between the two groups although less pronounced, decreasing from 0.22 ± 0.05 ml/cm^3^ to 0.18 ± 0.02 ml/cm^3^, indicating lower radiotracer binding in the respective tissue. Similarly, the Logan plot analysis of brain tissue presents a small decrease from 0.23 ± 0.05 ml/cm^3^ to 0.17 ± 0.02 ml/cm^3^ although not significantly different.

Additionally, the 2TCM was evaluated to account for potential receptor binding and internalization. It revealed a pronounced reduction in the binding-related rate constant k3 in tumor tissue following CXCR4 blockade (0.04 ± 0.02 vs 0.002 ± 0.004 min^−1^), while only minor changes were observed in the brain. Similarly, total distribution volume (Vt) decreased in tumor (0.52 ± 0.12 vs 0.28 ± 0.03), consistent with a CXCR4-specific binding component.

In contrast, k4 values exhibited high variability, particularly under blocking conditions, and transport parameters (K1, k2) showed moderate changes, indicating limited identifiability of individual rate constants. These findings suggest that while 2TCM captures a specific binding component, reliable separation of underlying kinetic processes remains challenging.

### Ex vivo metabolism study of [^68^Ga]Ga-TD-01

The stability of [^68^Ga]Ga-TD-01 was analyzed at various time points beyond the imaging protocol using radio-HPLC. The radiotracer extraction efficiency, using 0.1% TFA in methanol as extractant solvent, was shown to be higher than 95% of the total activity. [⁶⁸Ga]Ga-TD-01 remained predominantly intact during the scanning window (up to 60 min post-injection). At 30 min post-injection, a single major radioactive peak corresponding to the intact parent compound was observed, with no detectable secondary peaks indicative of radiolabeled metabolites. Similarly, at 60 min post-injection, the chromatographic profile remained unchanged, confirming the absence of measurable metabolic degradation over the imaging time window (Supplementary Fig. 3). On the other hand, significant radiotracer degradation was observed in the last two time points, ranging from 45% intact radiotracer at 90 min to 14% at 120 min. (Table 4). The retention time of the radioactive peak observed in the chromatograms matched that of the reference [^nat^Ga]Ga-TD-01 standard, while different early eluting time-dependent radiometabolites were observed at the latest investigated time points (Supplementary Fig. 7). These findings demonstrate the relative in vivo stability of [^68^Ga]Ga-TD-01, supporting its suitability for PET imaging and pharmacokinetic modeling. The absence of detectable radiometabolites during the imaging protocol timespan supports the validity of the derived tumor and organ time–activity curves and ensures that the PET signal predominantly reflects intact CXCR4-targeted ligand rather than other radioactive species.

**Table 4:** In vivo metabolism of [^68^Ga]Ga-TD-01

Time p.i. (min)	Intact tracer (%)	Number of experiments
30	> 99	n = 2
60	> 99	n = 2
90	45 ± 13	n = 3
120	14 ± 11	n = 3

## Discussion

Following the successful synthesis of [^68^Ga]Ga-TD-01 as presented recently by our group (Suwattananuruk et al. 2024), we aim to further evaluate this radiotracer as a potential tool for brain tumor imaging with PET in patients. Given its inhibitory potency at the CXCR4 receptor of IC_50_ = 36.5 nM we conducted in the current study receptor inhibition experiments with the highly specific receptor antagonist TIQ15 (IC_50_ = 7.5 nM (Suwattananuruk et al. 2024), 6.2 nM (Truax et al. 2013b)) using dynamic PET imaging and full pharmacokinetic modeling. Furthermore, we demonstrated the radiation safety of the radiotracer as a prerequisite for the approval of early clinical trials.

The pharmacokinetic profile of [^68^Ga]Ga-TD-01 is characterized by rapid blood clearance, low background uptake in most tissues, and predominant renal excretion, which are favorable properties for molecular imaging. When compared with established CXCR4-targeted radiotracers such as [^68^Ga]Ga-Pentixafor, [^68^Ga]Ga-TD-01 demonstrates a pharmacokinetic profile consistent with previously reported CXCR4-targeted radiotracers, including rapid systemic clearance and renal elimination. (Lindenberg et al. 2024).

Dynamic PET imaging presents clear and distinct uptake of [^68^Ga]Ga-TD-01 in the mouse brain tumor within the range of values reported in human GBM with [^68^Ga]Ga-Pentixafor (Jacobs et al. 2022a) which expresses similar binding affinity to CXCR4 (Schottelius et al. 2017). However, [^68^Ga]Ga-Pentixafor is presenting a large variety in tumor uptake, independent of CXCR4 expression status of the patient-specific GBM (Jacobs et al. 2022a), while consistent tumor uptake was observed across animals in the present study. Furthermore, reported uptake values for [^68^Ga]Ga-Pentixafor vary markedly across models, with high-CXCR4 lymphoma xenografts reaching about 16.2%ID/g, whereas lower-expression models have shown substantially lower uptake, for example about 3.5%ID/g in SU-DHL-8 xenografts (Wester et al. 2015). Further preclinical studies of CXCR4-targeted radiotracers report a broad range of tumor uptake values depending on tumor type and model. For example, Demmer et al. (Demmer et al. 2011) reported tumor uptake of approximately 6%ID/g for a CXCR4-targeted tracer in a mouse small-cell lung cancer xenograft model, with receptor specificity confirmed by blocking experiments. Building on this, Gourni et al. (Gourni et al. 2011) reported tumor uptake in the range of 4.55–6.16%ID/g using an optimized CXCR4-specific radiotracer in the same model. Although these ectopic tumor models are not directly comparable to orthotopic GBM, they provide a useful reference range, indicating that the uptake of [^68^Ga]Ga-TD-01 (approximately 5%ID/g) is consistent with values reported for CXCR4-targeted radiotracers in preclinical studies.

These comparisons indicate that the uptake of [^68^Ga]Ga-TD-01 is within the range reported for CXCR4-targeted tracers, but direct conclusions regarding superiority over [^68^Ga]Ga-Pentixafor are not justified because no head-to-head comparison was performed under identical experimental conditions. In addition, CXCR4 expression and [^68^Ga]Ga-Pentixafor uptake in GBM are known to be heterogeneous, and caution is warranted when extrapolating across tumor entities and study designs (Jacobs et al. 2022b).

The observed TBR in our study exceeds commonly used thresholds reported for amino acid PET tracers such as [^18^F]FET, although direct comparison across tracers should be interpreted cautiously (Zhao et al. 2020). Recommendations from expert panels like RANO (Albert et al. 2024) or ESTRO-EANO (Niyazi et al. 2023) recommend a TBR threshold of 1.6 for [^18^F]FET.

To investigate the specificity of this signal, receptor blocking studies with the highly affine TIQ15 were performed and showed a significant decrease of [^68^Ga]Ga-TD-01 in tumor tissue, supporting the presence of a CXCR4-specific binding component in line with previous reports of CXCR4 expression in GBM (Jacobs et al. 2022a; Ehtesham et al. 2006) and in particular in GL261 cells (Cornelison et al. 2018).

Although the absolute tumor uptake of [^68^Ga]Ga-TD-01 observed in this study is moderate compared with some previously reported CXCR4-targeted radiotracers, in the context of brain imaging, absolute uptake values must be interpreted together with background signal. Notably, [^68^Ga]Ga-TD-01 exhibited low uptake in normal brain tissue, resulting in a high tumor-to-background ratio, which is a key determinant of lesion detectability in neuro-oncology imaging. This high contrast suggests that even moderate tumor accumulation may be sufficient for sensitive tumor visualization. Furthermore, the observed reduction in tracer uptake and distribution volume upon CXCR4 blockade supports the presence of a specific binding component contributing to the imaging signal. Nevertheless, the relatively modest absolute uptake may limit applications requiring high absolute tumor retention, such as radionuclide therapy. Therefore, while the present data support the utility of [^68^Ga]Ga-TD-01 for high-contrast imaging of CXCR4 expression in GBM, further optimization or validation in additional models may be required to fully assess its therapeutic potential.

The RNA scope analysis presented in this study provides compelling evidence of the tumor-specific expression of CXCR4 in both murine and human GBM. The exclusive punctate staining observed in tumor regions, and the absence of signal in surrounding healthy brain tissue, reinforce the notion that CXCR4 is selectively upregulated in glioma cells. This spatially distinct expression pattern is critical for the development and application of targeted molecular imaging and therapeutic agents. In the mouse model, the GL261 tumors exhibited strong CXCR4 transcript signals, in line with prior reports of high CXCR4 expression in this cell line (Cornelison et al. 2018). Importantly, the parallel findings in human GBM samples confirm the translational relevance of this target and validate the use of GL261 as a preclinical model for CXCR4-directed imaging. Resultantly, the observed increase in tumor-to-brain RNAscope signal corresponded with the high tumor-to-background contrast detected by PET, supporting the interpretation that tracer accumulation is at least partially driven by target-specific CXCR4 expression. Nevertheless, further studies incorporating fully quantitative spatial correlation analyses between histological expression and PET-derived kinetic parameters would be valuable to more comprehensively define the relationship between receptor density and tracer uptake.

The in vitro findings were further confirmed by pharmacokinetic modeling with the 1TCM where Vt, the diffusion independent parameter (Logan et al. 1994), was significantly decreasing during blocking experiments due to an increase of k2 under pre-blocking conditions. Of note, blocking effects were observed in the healthy brain region as well although to a lesser extent. Pharmacokinetic modeling with the 1TCM confirmed lower Vt in the brain. The uptake in healthy brain under blocking conditions might be due to CXCR4 expression in healthy mouse brain tissue (data available from https://www.proteinatlas.org/ENSG00000121966-CXCR4/brain) shown in our model by physiological uptake of [^68Ga^]Ga-TD-01. Importantly, the consistency of Vt estimates obtained from Logan graphical analysis further supports the robustness of the kinetic interpretation independent of compartmental model assumptions. As Logan analysis does not rely on explicit separation of binding and internalization compartments, the agreement with compartmental modeling suggests that the contribution of receptor internalization cannot be reliably distinguished within the current dataset (Lubberink and Heurling 2019). Together, these findings indicate that increasing model complexity does not improve the biological interpretability of the data, supporting the use of the simpler 1TCM.

CXCR4 is a G-protein-coupled receptor known to undergo ligand-induced internalization (Yu et al. 2023), which in principle could give rise to more complex tracer kinetics than described by a 1TCM. In such a scenario, a 2TCM may represent reversible binding and subsequent internalization as separate compartments (Ringheim et al. 2020). Under baseline conditions, k3 values were higher in tumor compared with brain, and importantly, a significant reduction in both k3 and Vt was observed in tumor tissue following CXCR4 blockade, while no significant changes were detected in the brain. These findings support the presence of a CXCR4-specific binding component that can be captured by the 2TCM.

However, despite this sensitivity to receptor blockade, k4 values showed substantial variability, particularly under blocking conditions, and K1 and k2 exhibited non-physiological changes, indicating parameter coupling and limited identifiability. Thus, while the 2TCM provides supportive evidence for specific binding, the instability of individual rate constants limits its utility for robust kinetic quantification. Consequently, the 1TCM was considered the most reliable and parsimonious model for describing tracer kinetics under the present experimental conditions.

Of note, due to the significant lower radiotracer uptake in healthy brain compared to GBM tissue, the tumor was clearly visible in the PET as hyperintense region. These results demonstrate that [^68^Ga]Ga-TD-01 is capable of selectively targeting CXCR4-positive tumors while maintaining low background uptake in healthy brain regions. These characteristics are particularly important for GBM imaging, where tumor margins must be clearly delineated to guide treatment planning, including surgical resection and radiotherapy.

The binding affinity of [^68^Ga]Ga-TD-01 (IC_50_ ≈ 36 nM) falls within the moderate nanomolar range and is comparable to values reported for established CXCR4-targeted radiotracers such as [^68^Ga]Ga-Pentixafor, which typically exhibit affinities of approximately 25 nM (Schottelius et al. 2017; Poschenrieder et al. 2016a; Osl et al. 2020; Poschenrieder et al. 2016b). Importantly, binding affinity alone does not determine in vivo imaging performance. Rather, tracer uptake and image contrast are influenced by multiple factors, including receptor density, ligand internalization, plasma protein binding, and systemic pharmacokinetics (Innis et al. 2007; Mintun et al. 1984).

In the present study, the observed tumor uptake and favorable tumor-to-background ratios, despite moderate affinity, may reflect a combination of low nonspecific uptake in normal brain tissue and sufficient CXCR4 expression in the tumor model. Of note, very high binding affinity does not necessarily improve imaging performance, as optimal image contrast depends on a balance between receptor binding and favorable pharmacokinetic properties, including sufficient washout from non-target tissues (Laverman et al. 2012).

Overall, the imaging characteristics of [⁶⁸Ga]Ga-TD-01 likely reflect a balance between receptor binding and pharmacokinetic behavior. These findings support its suitability for CXCR4-targeted PET imaging, while further optimization of ligand affinity and structure–activity relationships remains an important direction for future studies.

The incorporation dosimetry in humans based on mouse biokinetic data revealed an ED of 12.5 μSv/MBq which is well in the range of other diagnostic PET radiotracers like [^18^F]FDG (19–20 μSv/MBq (Icrp 2008; Quinn et al. 2016)), [^68^Ga]Ga-Pentixafor (15.3 μSv/MBq (Herrmann et al. 2015)) or [^18^F]FET (16.5 μSv/MBq (Pauleit et al. 2003)) and equals 4.6 mSv for a standard PET scan with 370 MBq [^68^Ga]Ga-TD-01. However, as shown previously when using mouse biokinetic data for human dose estimation, an underestimation of up to 50% was reported by our group (Kranz et al. 2016a) and others (Garrow et al. 2020; Sakata et al. 2013). Resultantly, an ED following injection of 370 MBq of [^68Ga^]Ga-TD-01 into human subjects will cause a radiation burden of up to 6.9 mSv which is below the constraints set by several regulatory entities (ranging between 10 -30 mSv/study) (Commission and Environment 1998; Code and of Federal Regulations Title 21,2024; Protection ICoR 1999). As with other PET radiotracers (Garrow et al. 2020), the excretory organs are among those receiving the highest ODs and ED. In our study, urinary bladder wall and kidneys receive the highest OD and ED contribution, supporting recommendations for adequate hydration and frequent urination following radiotracer administration to reduce the absorbed doses to these organs. Whole-body dosimetry will need to be validated in first-in-human studies involving a limited number of healthy volunteers.

The predicted tumor dose for a hypothetical [^67^Cu]Cu-TD01 application was estimated to be 39.3 ± 15.2 mGy/MBq and per cycle. As monotherapy it is below what external radiotherapy achieves in current GBM treatment scenarios. Fractionated absorbed doses of up to 60 Gy are required to achieve prolonged patient survival (Walker et al. 1979). However, the total delivered tumor dose in a therapeutic setting would depend on the injected activity per cycle and the number of treatment cycles applied. Consequently, cumulative tumor doses may be substantially higher than the single-cycle estimates presented here. In our study, radiotoxicity to several organs limits the injected dose per cycle. Notably, although the extrapolated tumor dose estimates for [^67^Cu]Cu-TD-01 indicate potential therapeutic efficacy, it must be emphasized that these estimates are based exclusively on dosimetric modeling from the diagnostic analog [^68^Ga]Ga-TD-01. These estimates are in the range of our previous study using [^67^Cu]Cu-rhPSMA-10.1 (Ekaney et al. 2025). The absence of direct biodistribution and radiochemical validation of the therapeutic compound renders these dosimetric projections hypothesis-generating rather than predictive of therapeutic efficacy and further experimental validation of [^67^Cu]Cu-TD-01 remains essential to determine achievable tumor doses and therapeutic efficacy. Nevertheless, such pharmacokinetic-based dose modeling provides a useful framework for early-stage evaluation of radiopharmaceutical candidates and may guide future optimization strategies, including modification of ligand structure, dosing regimens, or combination with other treatment modalities. Future studies must include full radiolabeling and stability testing, as well as multi-time-point SPECT imaging, to confirm the therapeutic feasibility and safety profile of [^67^Cu]Cu-TD-01.

The translational potential of [^68^Ga]Ga-TD-01 should be interpreted in the context of its pharmacokinetic profile and imaging characteristics. In the present study, the tracer demonstrated high tumor-to-background contrast in an orthotopic glioblastoma model, primarily driven by very low nonspecific uptake in normal brain tissue. This feature is particularly relevant for neuro-oncological imaging, where background signal often limits lesion detectability and delineation.

Beyond absolute tumor uptake, these findings highlight the importance of contrast-driven imaging performance, especially in the central nervous system. The observed kinetics of [^68^Ga]Ga-TD-01, including rapid clearance from non-target tissues and stable tumor retention within the imaging window, support its suitability for PET-based visualization of CXCR4 expression.

Compared to existing CXCR4-targeted tracers, the performance of [^68^Ga]Ga-TD-01 appears to fall within the expected range reported for this class of compounds (Jacobs et al. 2022a; Serfling et al. 2022; Poschenrieder et al. 2016a; Herrmann et al. 2015; Lapa et al. 2016). In this context, the combination of moderate affinity and favorable pharmacokinetics may contribute to efficient target visualization without excessive background retention. These characteristics may be particularly advantageous in anatomical regions with inherently low tracer uptake, such as the brain.

Taken together, these findings suggest that [^68^Ga]Ga-TD-01 represents a promising candidate for CXCR4-targeted PET imaging, particularly in neuro-oncological applications, while further studies are required to define its role relative to established tracers.

Despite the encouraging results, this study has several limitations that should be addressed in subsequent work.; (i) A limited number of research animals was used. However, as the hypothesized effects with this number could be clearly and significantly shown and are hence regarded as sufficient. The animal numbers were estimated by an a priori power analysis (G*Power) which confirmed post hoc the achieved statistical power, (ii) The GL261 remains one of the most widely used immunocompetent GBM models that allows evaluation of tracer behavior in an immunocompetent brain environment. However, it does not fully reflect the molecular heterogeneity and immune landscape of human GBM, which may influence CXCR4 expression and tracer uptake. The presence of CXCR4 in both GL261 and human GBM tissue, as confirmed by RNAscope, supports the relevance of the target, although further validation in additional models is warranted. (iii) Instead of Monte Carlo simulation of the actual tumor VOI for tumor dosimetry, the widely applied sphere model was used as implemented in OLINDA (Stabin et al. 2005) and IDAC-dose 2.1 (Andersson et al. 2017). (iv) The input function for PET pharmacokinetic modeling was derived from imaging and not by invasive measurement due to the complexity of experiments and the accessibility of the animal in the PET/MRI bore. However, the vena cava has been shown to be a suitable model for collecting data of the image derived input function (Espedal et al. 2021; Lanz et al. 2014) and hence was chosen here. Furthermore, the primary conclusions of this study rely on relative comparisons between baseline and blocking conditions, as well as consistency across modeling approaches (1TCM, 2TCM, and Logan analysis), which are less sensitive to systematic biases in the input function. The observed reductions in tumor uptake and distribution volume upon CXCR4 blockade were consistent across methods, supporting the robustness of the findings despite potential limitations in absolute quantification. (v) Since radiotracer metabolism is primarily driven by systemic processes, stability assessment in healthy animals is generally considered sufficient to evaluate tracer integrity during the imaging time window, although tumor-specific effects cannot be completely excluded. (vi) Although CXCR4 is known to undergo ligand-induced internalization, the 2TCM analysis did not provide stable or biologically meaningful estimates of the additional rate constants (k3 and k4). This is likely due to limited identifiability given the temporal resolution and signal-to-noise ratio of the data. Consequently, the simpler 1TCM was preferred based on robustness and model selection criteria. These findings suggest that, within the current experimental conditions, tracer kinetics can be adequately described without explicitly modeling an internalized compartment. (vii) While receptor blocking and RNAscope analyses confirmed CXCR4 specificity, off-target binding in other CXCR4-expressing organs and the potential impact of inflammatory microenvironments were not systematically evaluated. (viii) Furthermore, although dosimetric calculations using allometric scaling from mice to humans is a well-established methodology, it is inherently limited by interspecies differences in physiology, metabolism, and organ clearance. These differences can significantly influence biodistribution and radiation burden. As previously reported in our own studies (Kranz et al. 2016a; Kranz et al. 2016b) and by others (Zanotti-Fregonara and Innis 2012; Zanotti-Fregonara et al. 2013), such extrapolations may underestimate human absorbed dose by up to 50%. Therefore, dedicated human dosimetry studies will be essential to confirm these preclinical findings before clinical translation of [⁶⁸Ga]Ga-TD-01 or its therapeutic counterpart.

## Conclusions

This study demonstrates that TD-01 represents a promising CXCR4-targeting molecular scaffold with favorable pharmacokinetic and binding characteristics for application in GBM. Using quantitative PET as a non-invasive pharmacological tool, we show that [^68^Ga]Ga-TD-01 exhibits high in vivo stability, evidence of CXCR4-mediated tumor uptake, and low nonspecific accumulation in healthy brain tissue. Receptor blocking and compartmental pharmacokinetic modeling confirmed target engagement and competitive inhibition at the CXCR4 receptor in vivo.

Whole-body dosimetry estimates indicate an acceptable radiation burden for clinical PET imaging, supporting further clinical evaluation of [^68^Ga]Ga-TD-01 as a diagnostic agent. Importantly, PK-guided extrapolation of tumor time–activity data provides a quantitative rationale for the development of a matched copper-67–labeled therapeutic analog. The predicted absorbed tumor dose estimates suggest that [^67^Cu]Cu-TD-01 may support future evaluation for targeted radionuclide therapy of CXCR4-expressing GBM while preserving the molecular identity and pharmacological behavior of the diagnostic compound.

While the therapeutic dosimetry remains predictive and requires direct experimental validation, this work provides a framework for theranostic development based on quantitative pharmacokinetics rather than qualitative imaging alone. The integration of ligand design, target validation in human tumor tissue, in vivo pharmacology, and dosimetric modeling highlights the value of PET-guided drug development strategies for precision oncology.

Collectively, these findings support the continued development of the TD-01 platform as a CXCR4-directed candidate for GBM imaging and provide a basis for future studies focusing on therapeutic radiolabeling, biodistribution, efficacy, and safety evaluation in preparation for first-in-human translation.

## Supplementary Information

Additional file1 (PDF 1461 KB)

## Data Availability

The datasets used and/or analyzed during the current study are available from the corresponding author on reasonable request.

## Abbreviations

AIC: Akaike information criterion
ANOVA: Analysis of variance
CXCR4: CXC chemokine receptor 4
DOTA: 1,4,7,10-Tetraazacyclododecane-1,4,7,10-tetraacetic acid
ED: Effective dose
FET: O-(2-[^18^F]fluoroethyl)-L-tyrosine
FFPE: Formalin-fixed paraffin-embedded
GBM: Glioblastoma multiforme
H&E: Hematoxylin and eosin
HPLC: High-performance liquid chromatography
IDIF: Image-derived input function
1TCM: One-tissue compartment model
2TCM: Two-tissue compartment model
MRI: Magnetic resonance imaging
MSC: Model selection criterion
NOD: Number of disintegrations
OD: Organ dose
PET: Positron emission tomography
PET/MRI: Simultaneous positron emission tomography and magnetic resonance imaging
RNAscope: RNA in situ hybridization assay
SC: Schwarz criterion
SD: Standard deviation
SPE: Solid-phase extraction
TAC: Time–activity curve
TBR: Tumor-to-background ratio
VOI: Volume of interest
Vt: Volume of distribution
%ID: Percentage of injected dose
