# Supplementary material for: CXCR4-targeted PET imaging of glioblastoma using [68Ga]Ga-TD-01: from pharmacokinetics and dosimetry to theranostic potential
Source: EJNMMI Radiopharm Chem. 2026 May 21;11:46. doi: 10.1186/s41181-026-00457-9 (PMC13365296; doi:10.1186/s41181-026-00457-9)
Supplement: Supplementary file 1 — Additional file1 (PDF 1461 KB) [file 41181_2026_457_MOESM1_ESM.pdf]

Supplement: CXCR4-Targeted PET Imaging of Glioblastoma Using [^{68}Ga]Ga-TD-01: From Pharmacokinetics and Dosimetry to Theranostic Potential

Table of Contents

1. **Radiochemistry** 2
   1.1 Radio labeling and quality control of [^{68}Ga]Ga-TD-01 2
2. **PET/MR image segmentation** 6
3. **Power analysis** 6
4. **Whole-body organ dosimetry** 7
5. **PET pharmacokinetic modeling** 9
6. **Ex vivo stability** 10
7. **RNA scope group comparison** 11

1. Radiochemistry

1.1 Radio labeling and quality control of [^{68}Ga]Ga-TD-01

The BFC containing DOTA for ^{68}Ga complexation was successfully synthesized. Radiolabeling with ^{68}Ga was performed via a 10-minute reaction at 60 °C in sodium acetate solution (pH 4.0). The crude product was purified by solid phase extraction (HLB SPE Cartridge) to remove unreacted ^{68}Ga. Consequently, this condition (Fig. 1) was selected for labeling TD-01 and conducting quality control of the [^{68}Ga]Ga-TD-01 complex. The radio-TLC and HPLC chromatograms confirmed the successful radioactive labeling of ^{68}Ga with TD-01 (Fig. 2 and 3). After removing excess Ga-68 using an HLB SPE cartridge, [^{68}Ga]Ga-TD-01 exhibited radiochemical purity (RCP) exceeding 97% (n = 4). The calculated molar activity of the produced [^{68}Ga]Ga-TD-01 was 51.14±4.74 GBq/µmol. The quality control analysis results of [^{68}Ga]Ga-TD-01 for CXCR4 imaging are reported in Table 1.

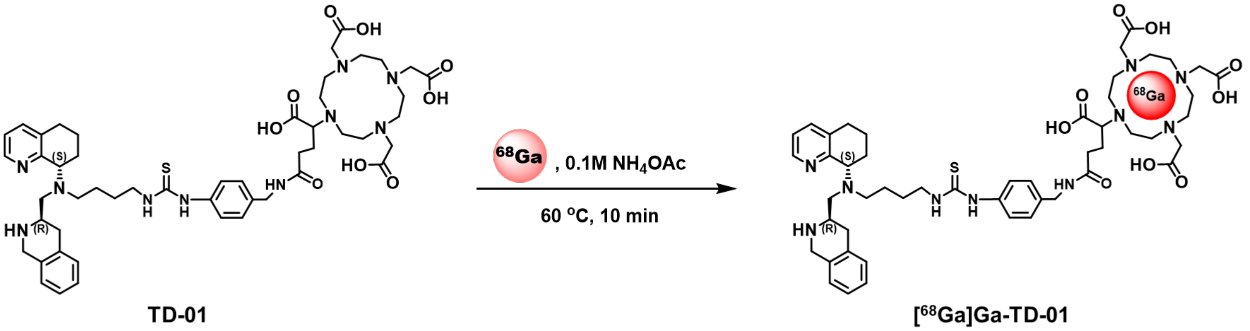

Figure 1 Radiosynthesis of [^{68}Ga]Ga-TD-01.

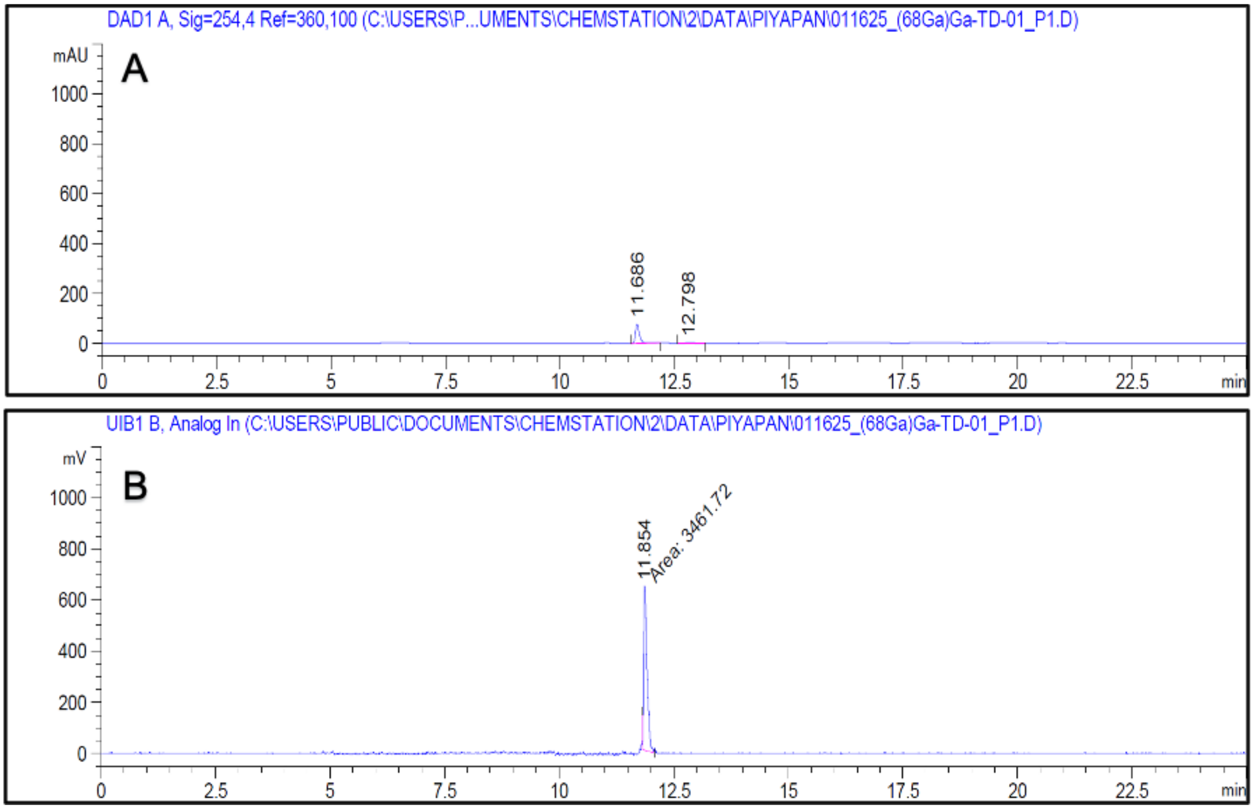

Figure 2: *HPLC chromatogram of [^{68}Ga]Ga-TD-01. (A) UV-detector and (B) Radio-detector*

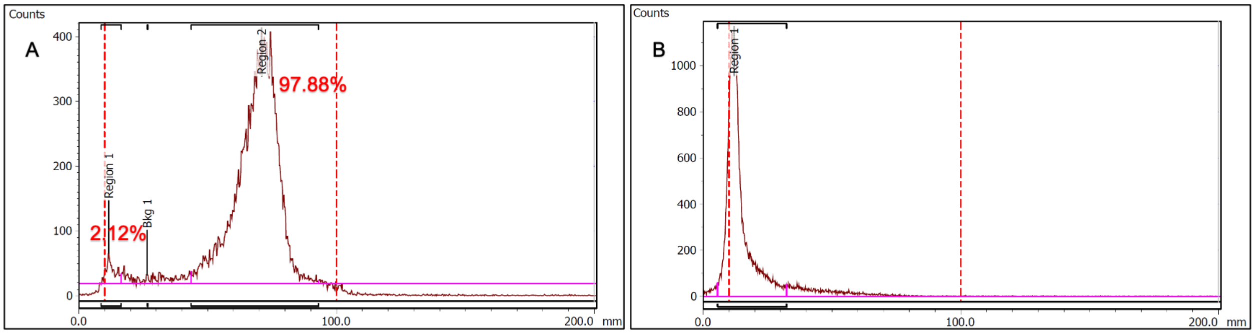

Figure 3: *iTLC profile of [^{68}Ga]Ga-TD-01. (A) 0.1 M NH_4OAc : MeOH = 4:6 and (B) 100% of sodium citrate.*

Table1: ***Shelf life of [^{68}Ga]Ga-TD-01 in normal saline at 1, 2, 3, 4, 5 and 6 hours (n = 3)***

Time (h)	HPLC chromatogram	%RCP
1	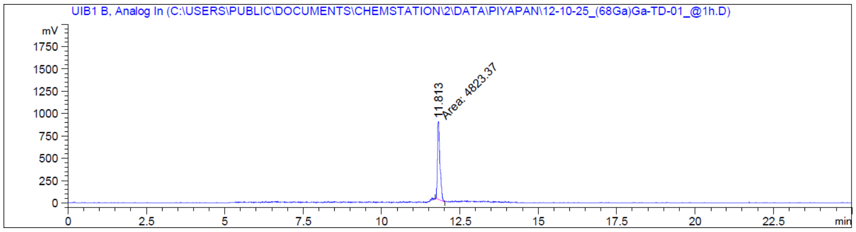	> 99

**2**	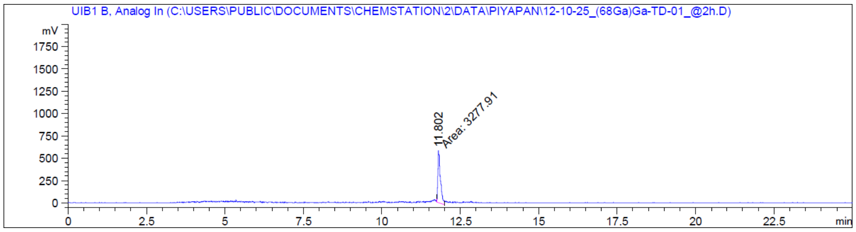	**> 99**
**3**	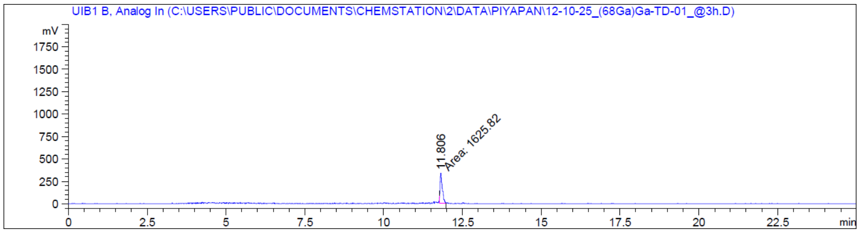	**> 99**
**4**	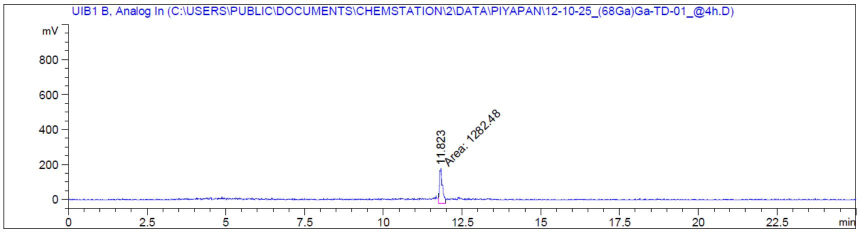	**> 99**
**5**	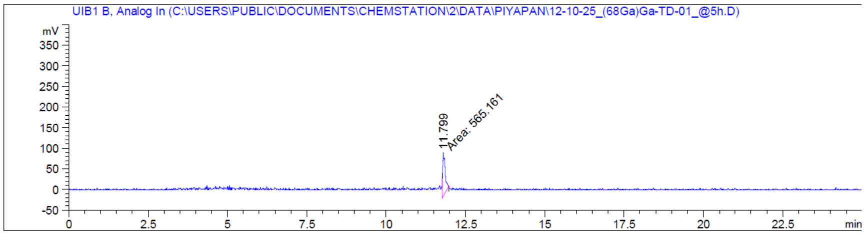	**> 99**
**6**	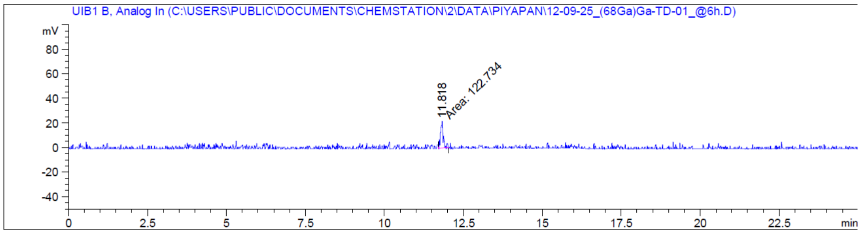	**> 99**

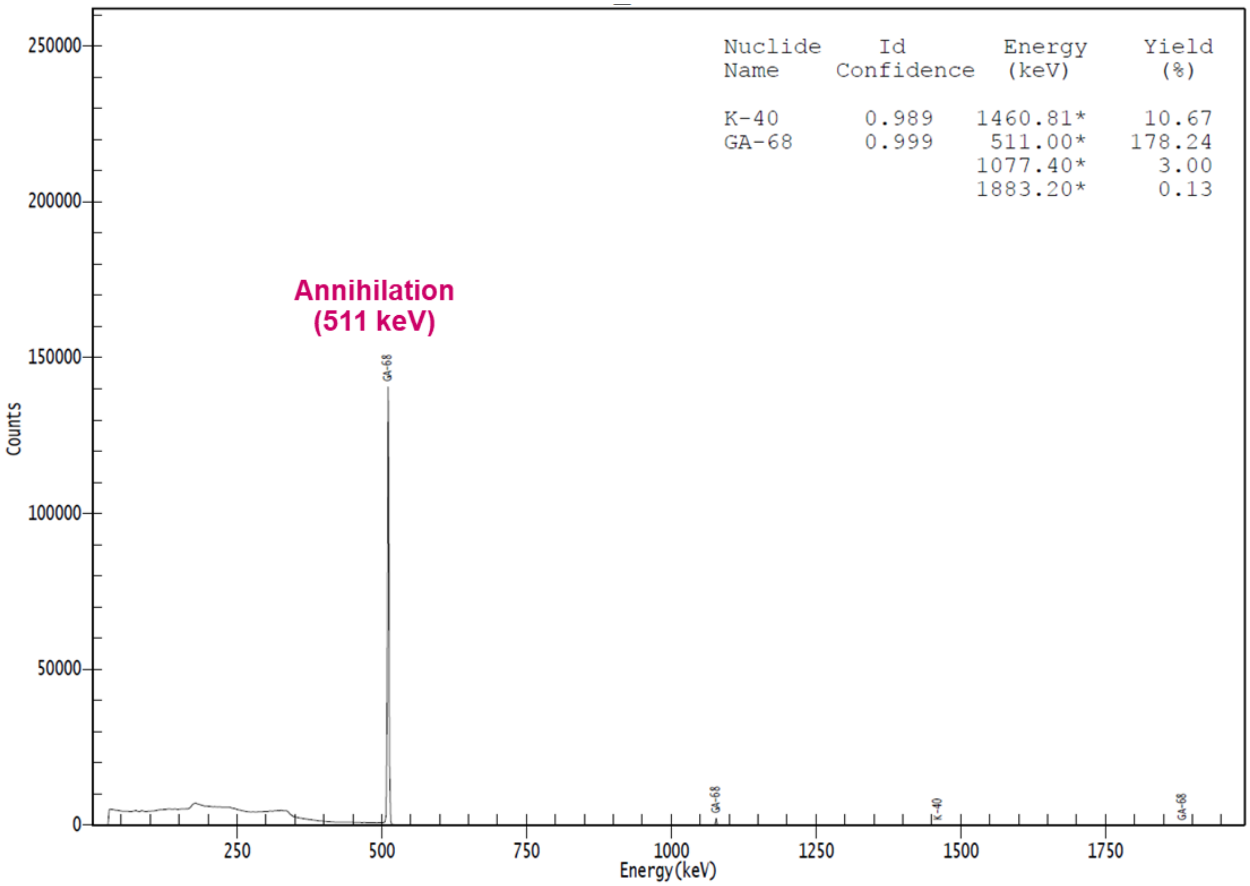

Figure 4: Gamma spectrum of Ga-68 sample. The gamma spectrum displays the energy distribution of counts obtained from a Ga-68 sample. The measurement was conducted over a live time of 3496.241 seconds and a real time of 3600.000 seconds. The prominent peak at approximately 511 keV corresponds to Ga-68. Additional smaller peaks are also identified at various energies. The spectrum indicates a high radionuclidic purity for Ga-68, as evidenced by the dominant peak at the expected energy for Ga-68 emissions.

2. PET/MR image segmentation

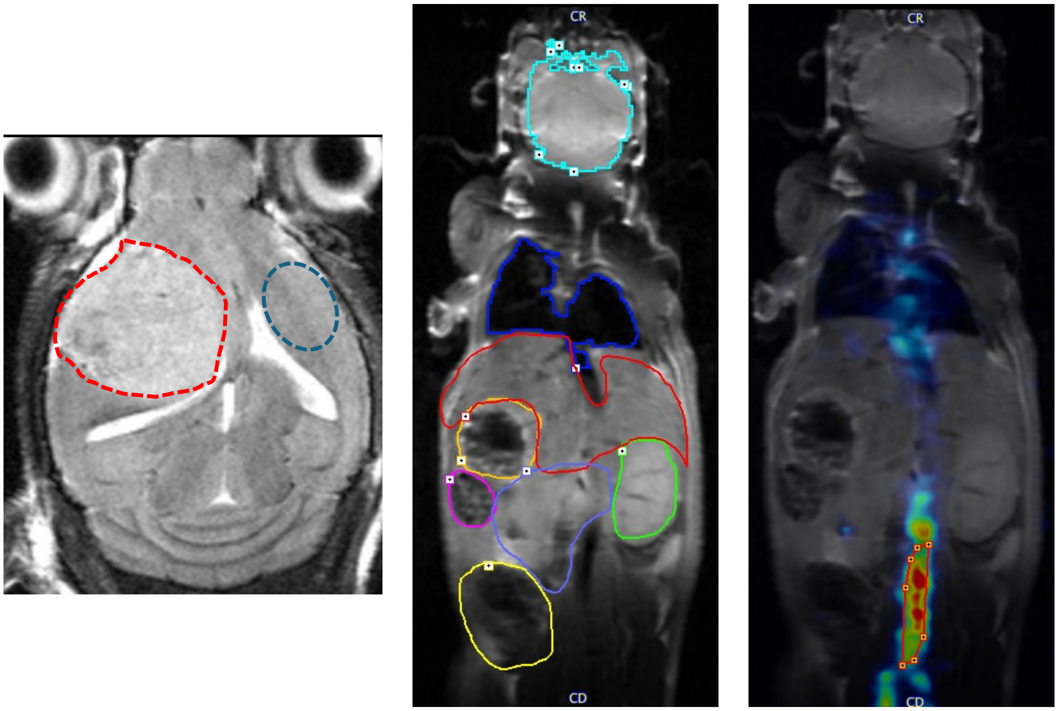

Figure 5: Examples of MRI based organ/VOI segmentation for left:T2-weighted FSE MRI, the tumor region (red) and contra lateral healthy brain region (blue) or middle: T1-weighted FSE MRI and whole body organ segmentation to extract time-activity data of dose relevant organs. Here brain (light blue), lungs (dark blue), liver (red), stomach (orange), spleen (magenta), right kidney (green) and small intestines (yellow) is visible. Right: Segmentation of the inferior vena cava (red) 5 s after radiotracer injection to extract the imaged derived input function for subsequent pharmacokinetic modeling.

3. Power analysis

<u>A priori: baseline vs. blocking tumor study</u>

t tests - Means: Difference between two independent means (two groups)

Analysis: A priori: Compute required sample size
Input: Tail(s) = Two
Effect size d = 2,7
α err prob = 0,05
Power (1-β err prob) = 0,95
Allocation ratio N2/N1 = 1
Output: Noncentrality parameter δ = 4,2690748
Critical t = 2,3060041
Df = 8
Sample size group 1 = 5
Sample size group 2 = 5
Total sample size = 10
Actual power = **<u>0,9606290</u>**

<u>Post hoc: baseline vs. blocking tumor study</u>

t tests - Means: Difference between two independent means (two groups)

Analysis:	Post hoc: Compute achieved power			
Input:	Tail(s)	=	Two	
	Effect size d	=	2,85	
	α err prob	=	0,05	
	Sample size group 1	=	6	
	Sample size group 2	=	6	
Output:	Noncentrality parameter δ	=	4,9363448	
	Critical t	=	2,2281389	
	Df	=	10	
	Power (1-β err prob)	=	**0,9931020**	

A post hoc power analysis was performed in G*Power 3.1 for the primary pharmacokinetic endpoint (tumor Vt, 1TCM). Based on the observed means and standard deviations (0.541 ± 0.065 vs. 0.325 ± 0.085), the standardized effect size was $d = 2.85$. With $\alpha = 0.05$ and $n = 6$ animals per group, the achieved power was >0.95.

4. Whole-body organ dosimetry

Table 2: OD, ED contributions and the ED based on mouse biokinetic data extrapolated to human entity and estimated with OLINDA 1.1 for the adult male model.

	**OD (µSv/MBq)**		**ED contr (µSv/MBq)**	
**Adult male (73.7 kg)**	mean	stdev	mean	stdev
**Adrenals**	9.82E-03	1.28E-03	7.58E-05	3.17E-05
**Brain**	5.29E-03	3.00E-03	4.53E-05	3.25E-05
**Breasts**	7.49E-03	1.79E-03	8.41E-04	3.61E-04
**Gallbladder Wall**	1.31E-02	1.52E-03	9.08E-05	5.38E-05
**LLI Wall**	1.31E-02	4.90E-03	1.07E-03	8.61E-04
**Small Intestine**	2.52E-02	1.59E-02	1.57E-04	1.91E-05
**Stomach Wall**	1.18E-02	1.07E-03	1.40E-03	1.10E-04
**ULI Wall**	2.08E-02	1.20E-02	7.92E-04	3.90E-04
**Heart Wall**	2.60E-02	2.54E-02	1.30E-04	3.40E-05
**Kidneys**	5.01E-02	2.32E-02	6.58E-04	7.58E-04
**Liver**	1.82E-02	1.17E-02	5.90E-04	3.67E-04
**Lungs**	2.45E-02	3.14E-02	1.35E-03	5.56E-04
**Muscle**	9.51E-03	1.87E-03	6.83E-05	2.40E-05
**Ovaries**	1.18E-02	3.77E-03	1.42E-03	1.25E-03
**Pancreas**	9.68E-03	1.73E-03	7.63E-05	3.37E-05
**Red Marrow**	7.84E-03	1.08E-03	9.59E-04	1.41E-04
**Osteogenic Cells**	1.38E-02	3.37E-03	1.41E-04	3.38E-05
**Skin**	8.53E-03	2.16E-03	8.72E-05	2.22E-05
**Spleen**	1.17E-02	4.02E-03	8.63E-05	4.80E-05

**Testes**	1.00E-02	3.05E-03	0.00E+00	0.00E+00
**Thymus**	8.44E-03	1.85E-03	6.58E-05	3.12E-05
**Thyroid**	9.72E-03	2.49E-03	4.27E-04	1.56E-04
**Urinary Bladder Wall**	5.15E-02	2.52E-02	2.08E-03	9.50E-04
**Uterus**	1.24E-02	3.45E-03	8.78E-05	2.74E-05
**Total Body**	8.95E-03	2.47E-03	0.00E+00	0.00E+00
**ED**	-	-	**1.25E-02**	**8.16E-04**

5. PET pharmacokinetic modeling

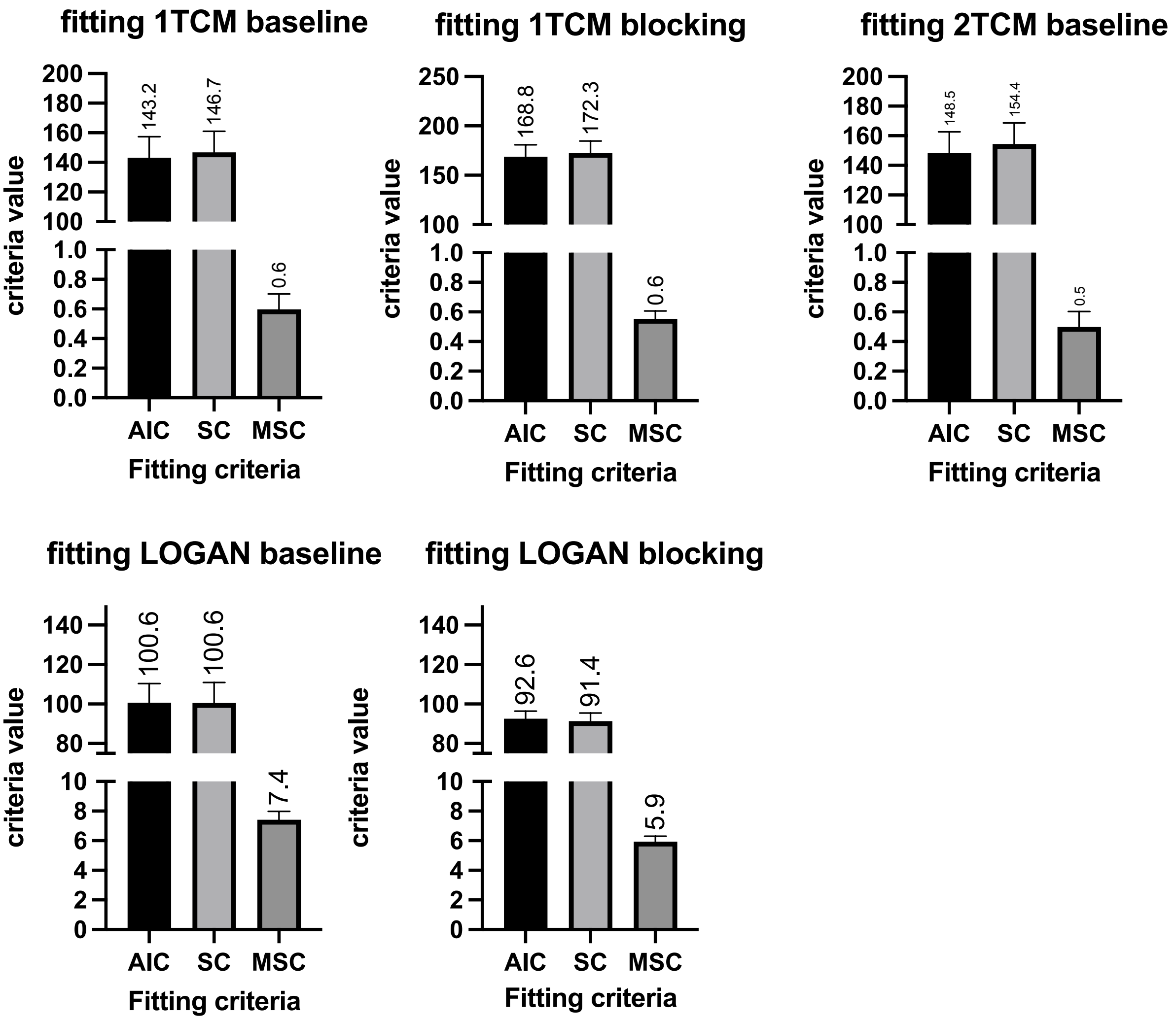

Figure 6: Evaluation of model fitting criteria using Akaike Information Criterion (AIC), Schwartz Criterion (SC) and Model Selection Criterion (MSC).

6. Ex vivo stability

Time (min)	HPLC chromatogram / gamma counter signal	Intact tracer (%)
30	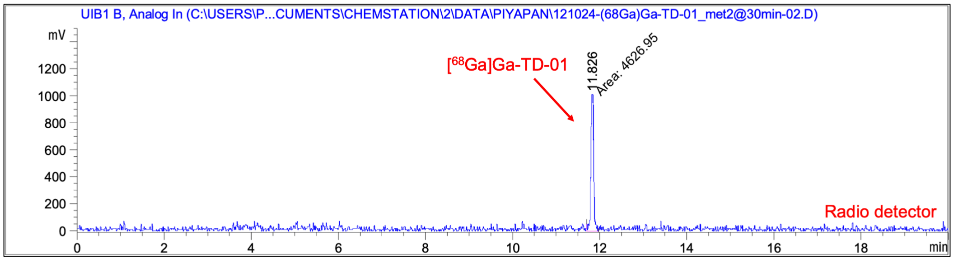	> 99
60	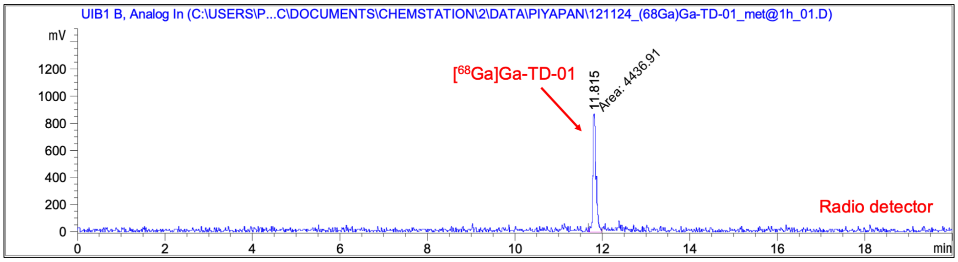	> 99
90	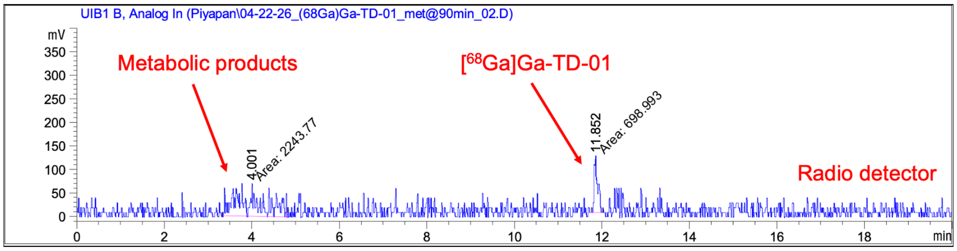	45 ± 13
120	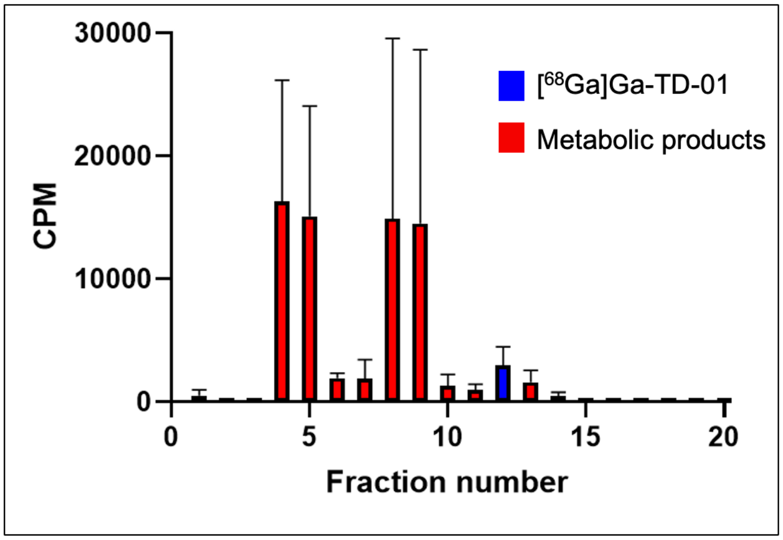	14 ± 11

Figure 7: Radio-HPLC chromatogram and gamma counter fractions of [^{68}Ga]Ga-TD-01 in the extracted supernatant at 0.5, 1, 1.5 and 2-hours post-injection.

7. RNA scope group comparison

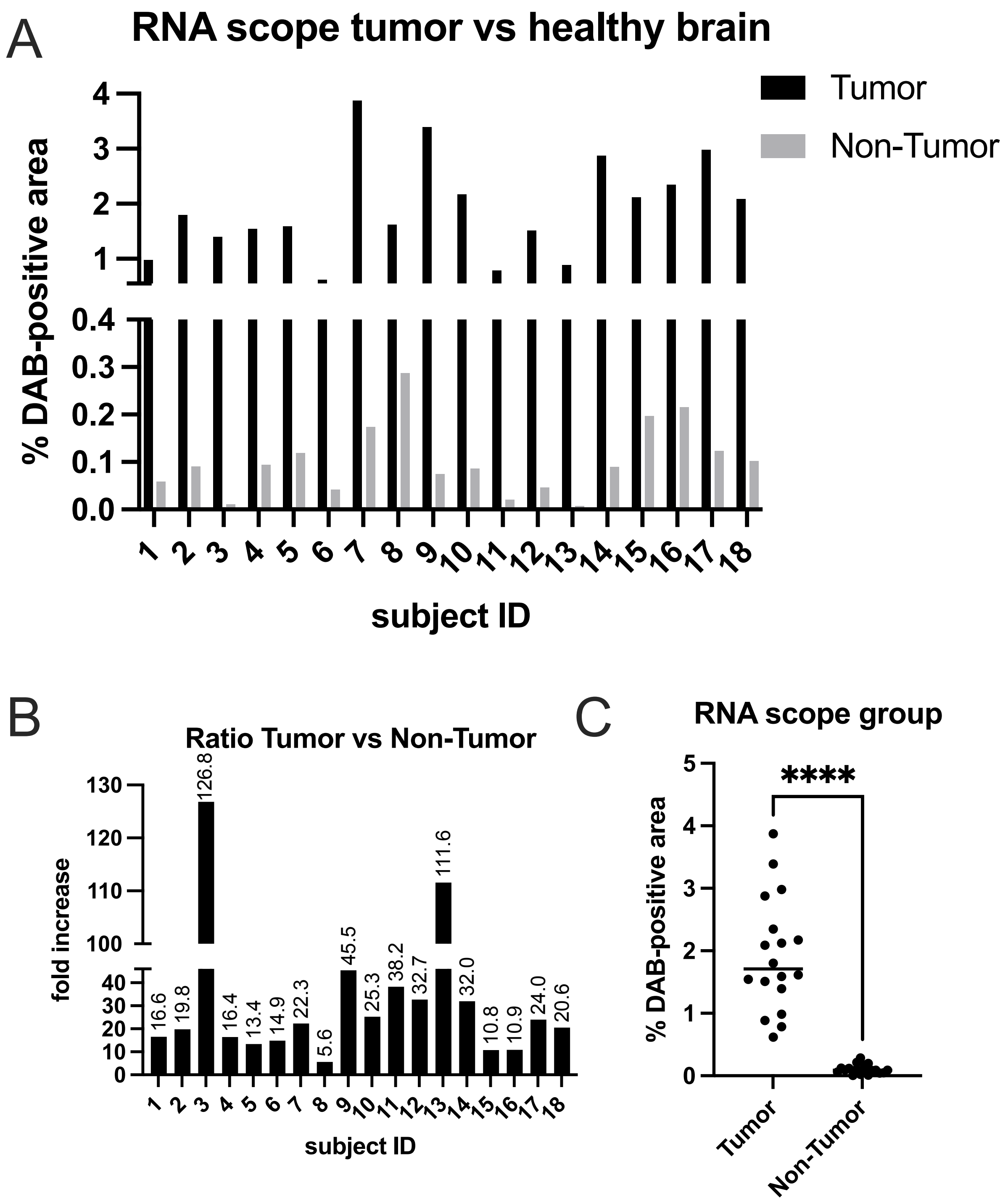

Figure 8: Quantitative analysis of CXCR4 RNAscope signal in orthotopic GL261 glioblastoma tissue and contralateral healthy brain. (A) Quantification of RNAscope-derived DAB-positive area in tumor and non-tumor brain regions for individual animals (n = 18). Tumor tissue demonstrated consistently higher CXCR4-associated signal compared to contralateral healthy brain tissue. (B) Tumor-to-brain fold increase in RNAscope signal intensity for each subject, illustrating elevated CXCR4 expression within the tumor microenvironment relative to normal brain tissue. (C) Grouped

analysis of RNAscope DAB-positive area (two-sided, unpaired t test) demonstrating significant increased CXCR4 expression in tumor tissue compared to non-tumor brain regions. Data are presented as mean ± SD.
